# High-fat diet induced cyclophilin B enhances STAT3/lncRNA-PVT1 feedforward loop and promotes growth and metastasis in colorectal cancer

**DOI:** 10.1038/s41419-022-05328-0

**Published:** 2022-10-20

**Authors:** Hanqing Guo, Kun Zhuang, Ning Ding, Rui Hua, Hailing Tang, Yue Wu, Zuyi Yuan, Ting Li, Shuixiang He

**Affiliations:** 1 First Affiliated Hospital, Xi’an Jiaotong University, Xi’an, China; 2 Department of Gastroenterology, Xi’an Central Hospital, College of Medicine, Xi’an Jiaotong University, Xi’an, China; 3 Department of Cardiovascular Diseases, First Affiliated Hospital of Xi’an Jiaotong University, Xi’an, China

**Keywords:** Long non-coding RNAs, Colorectal cancer

## Abstract

High-fat diet (HFD) has been implicated to promote colorectal cancer (CRC). Recently, oncogene Cyclophilin B (CypB) is reported to be induced by cholesterol. However, the role of CypB in CRC carcinogenesis and metastasis associated with HFD remains unknown. In the present study, we showed that HFD-induced CypB enhances proliferation and metastasis through an inflammation-driven circuit, including Signal Transducer and Activator of Transcription 3 (STAT3)-triggered transcription of lncRNA-PVT1, and its binding with CypB that promotes activation of STAT3. CypB was found to be upregulated in CRC, which was correlated with elevated body mass index and poor prognosis. HFD induced CypB expression and proinflammatory cytokines in colon of mice. Besides, CypB restoration facilitated growth, invasion and metastasis in CRC cells both in vitro and in vivo. Moreover, RIP sequencing data identified lncRNA-PVT1 as a functional binding partner of CypB. Mechanistically, PVT1 increased the phosphorylation and nuclear translocation of STAT3 in response to IL-6, through directly interaction with CypB, which impedes the binding of Suppressors Of Cytokine Signalling 3 (SOCS3) to STAT3. Furthermore, STAT3 in turn activated PVT1 transcription through binding to its promoter, forming a regulatory loop. Finally, this CypB/STAT3/PVT1 axis was verified in TCGA datasets and CRC tissue arrays. Our data revealed that CypB linked HFD and CRC malignancy by enhancing the CypB/STAT3/PVT1 feedforward axis and activation of STAT3.

## Introduction

Colorectal cancer (CRC), a multistep disease in which accumulated genetic and epigenetic alterations were driven by inflammation, is the third leading cause of new cancer cases and the second leading cause of cancer-related deaths worldwide [1, 2]. Strong associations between a sedentary lifestyle and obesity with CRC were conducted in both clinical and epidemiological studies [3, 4]. Recent studies suggest that CRC tumorigenesis is initiated by high-fat diet (HFD)-induced proinflammatory cytokines such as interleukin-6 (IL-6) and exacerbated by subsequent inflammatory burden [5, 6].

As the classic intracellular IL-6 signaling transductor, Signal Transducer and Activator of Transcription 3 (STAT3) regulates the expression of a variety of genes in response to inflammatory stimuli [3]. As a point of convergence for numerous oncogenic signaling pathways, excessive STAT3 activation within cancer cells can be viewed as a neoplastic mimic of an inflammation-driven repair response that collectively promotes tumor progression [7]. Extracellular binding of cytokines such as IL-6 to their cognate receptors induce activation of the intracellular Janus kinase (JAK) that phosphorylates a specific tyrosine residue in the STAT3 protein [8]. Once phosphorylated, STAT3 form homo-or hetero-dimers through interactions of phosphorylated tyrosine of one STAT and SH2 domain of another, then translocate into the nucleus [7, 9]. Unrestrained STAT3 activation is one of the hallmarks of CRC, contributing to its progression. It’s also considered that activation of STAT3 maintained obesity-related metastatic growth of CRC cells [10, 11]. However, the regulatory mechanism supporting the cascade amplification of IL-6/STAT3 pathway related to obesity in CRC is still not fully elucidated.

Recent studies have suggested that Cyclophilin B (CypB) overexpression is crucial in supporting the activation of STAT3 in tumors [12–16]. CypB is an endoplasmic reticulum (ER)-resident protein with peptidyl-prolyl cis/trans-isomerase activity (PPIase) [17], which belongs to a conserved protein family Cyclophilin, expressed ubiquitously in prokaryotic and eukaryotic organisms [18]. CypB is associated with the malignant progression and regulation of a variety of tumors [12–14, 19, 20]. We have reported functions of CypB in proliferation and survival of stomach cancer [14], demonstrating a supporting role of CypB in constitutive activation of IL-6/STAT3 pathway. Interestingly, several recent studies suggest that cholesterol induced CypB participated in initiation of metabolic syndrome and lung cancer [21, 22], suggesting a possible role of CypB connecting obesity with cancer. To date, few studies have elucidated CypB’s function on HFD-associated CRC proliferation and metastasis. Besides, although CRC is among several solid tumors that are characterized by constitutively STAT3 activation, which is closely connected with oncogenic CypB, the relationship of CypB and STAT3 activation in CRC remains largely unclear.

Long noncoding RNAs (lncRNAs) are a class of highly multifunctional noncoding RNAs (ncRNAs) larger than 200 nucleotides in size that lack coding potential [23], which were recently found to contribute to CRC tumorigenesis [24]. As several studies found that translocation of CypB from cytoplasm to nuclei where lots of functional lncRNA were synthesized, it’s reasonable to elucidate if CypB’s function is linked to any lncRNAs in CRC. However, few study have focused on the connection between CypB and lncRNAs. Here we present evidence that CypB/STAT3/lncRNA-PVT1 feedback loop triggered by HFD-associated IL-6 regulates progression of CRC. Our study found that HFD induced overexpression of CypB is required for STAT3 activation in the proliferation, survival and metastasis of CRC. We found that lncRNA-PVT1, binding directly to CypB, is transcriptionally promoted by STAT3, thus forming a feedforward circuit which potentially explain HFD-associated inflammation and subsequent IL-6-stimulation-triggered constitutive activation of STAT3 in CRC tumorigenesis.

## Results

### CypB is upregulated in CRC and predicted poor survival

**Fig. 1:**
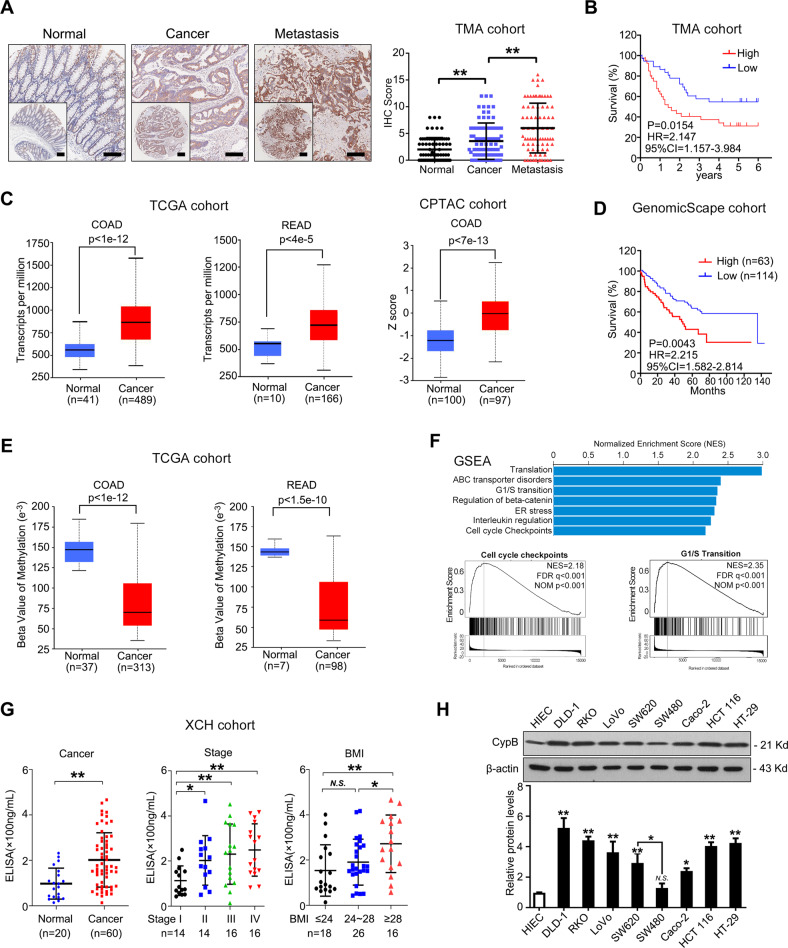
CypB is upregulated in CRC and predict poor survival. **A** Left: Representative images of IHC staining of CypB in a CRC tissue microarray containing paired tissue of primary tumors, adjacent normal tissues and metastatic lymph nodes (*n* = 80). Scale bars: 200 μm (main) and 500 μm (inset); Right: IHC scores of tissue arrays. **B** Kaplan-Meier curves of CRC patients of TMA cohort with low vs high expression of CypB (*n* = 80; *P* = 0.0154, HR = 2.147, 95% CI = 1.157–3.984). **C** Left: CypB levels in TCGA data of colon adenocarcinoma (COAD, *P* < 1e-12) and rectal adenocarcinoma (READ, P < 4e-5). Right: CypB levels in CPTAC data of COAD (*P* < 7e-13). **D** Kaplan-Meier curves of CRC patients with low vs high expression of CypB (*n* = 286; *P* = 0.0657, HR = 1.485, 95% CI = 0.951–2.169). **E** DNA methylation levels of CypB promoter of COAD and READ in TCGA cohorts. **F** Integrative GSEA analysis showing NES of CypB towards pathways in TCGA COAD and READ datasets (top). Enrichment plot of cell cycle checkpoints and G1/S transition (bottom). **G** Left: CypB levels in serum derived from healthy volunteers (*n* = 20) and CRC patients (*n* = 60). Middle: The levels of CypB in serum from CRC patients were divided by tumor stage. Right: CypB levels in serum derived from CRC patients were divided by BMI. **H** Immunoblot for CypB in 8 human CRC cell lines and HIEC cells. The data are presented as the means ± SDs. *P < 0.05; **, *P* < 0.01; N.S. not significant.

To examine the significance of CypB in CRC development, we first measured CypB expression in a cohort of 240 CRC samples (TMA cohort) using immunohistochemistry (IHC). CypB was significantly upregulated in CRC tissues and metastatic lymph nodes compared with adjacent non-cancerous colorectal tissues (Fig. 1A, Table S1). Correlation analysis revealed that higher level of CypB expression in CRC tissues was significantly associated with a more aggressive tumor phenotype (Table S2). Kaplan–Meier analysis further revealed that high-level CypB expression was associated with shorter disease-free survival time for CRC patients (Fig. 1B). Cox regression analysis also indicated that high CypB expression was an independent prognostic factor for poor survival in CRC patients (Table S3). Besides, we analyzed the expression of CypB in tissues of colon adenocarcinoma (COAD) and rectal adenocarcinoma (READ) based on the TCGA cohort and CPTAC cohort and found similar results (Fig. 1C), suggesting that both mRNA and protein levels are upregulated in CRC tissues. Similarly, pan-cancer analysis of CypB using TCGA data was conducted and CypB transcripts were found to be increased in 20 out of 31 kinds of cancer types, particularly gastrointestinal cancers such as ESCA (Esophageal carcinoma), STAD (Stomach adenocarcinoma), COAD and READ (Supplementary Fig. S1A). Further Kaplan–Meier analysis based on GenomicScape database [25] revealed that high-level CypB expression were associated with shorter disease-free survival time for CRC patients (Fig. 1D). TCGA data also showed that methylation levels of CypB promoter were decreased compared with that in normal tissues (Fig. 1E), possibly accounting for the overexpression of CypB in CRC. Further integrative analysis of Gene Sets Enrichment Analysis (GSEA) showed that CypB expression is positively associated with genes enriched in pathways of cell cycle, ER stress and regulation of Interleukin regulation and β-catenin (Fig. 1F, supplementary Fig S1B–D), which play important roles in the regulation of tumor proliferation and metastasis.

Moreover, as CypB can be secreted into serum, we measured CypB expression in sera from 60 CRC patients and 20 healthy volunteers. ELISA analysis indicated that the CypB concentrations in serum samples from CRC patients were significantly increased compared with those from volunteers (Fig. 1G). Interestingly, increased serum CypB concentrations were associated with tumor stage and BMI (Fig. 1G), indicating that overexpression of CypB may mediate the effects of high fat diet on the malignancy of CRC. Further, CypB expression was assayed in several CRC cell lines. Western blotting revealed that CypB expression was greater in several CRC cell lines compared with HIEC cells, an immortalized intestinal epithelial cell line (Fig. 1H). Compared with its expression in SW480 cells, CypB in SW620 cells is higher, suggesting its potential role in metastasis as SW620 cells are derived from the same patient’s metastatic lymph nodes as SW480. Together, these results suggest that CypB upregulation, occurring during CRC development, was associated with obesity and may have a vital role in colorectal carcinogenesis.

### HFD-induced CypB increases CRC cell growth, invasion, and metastasis in vitro

**Fig. 2:**
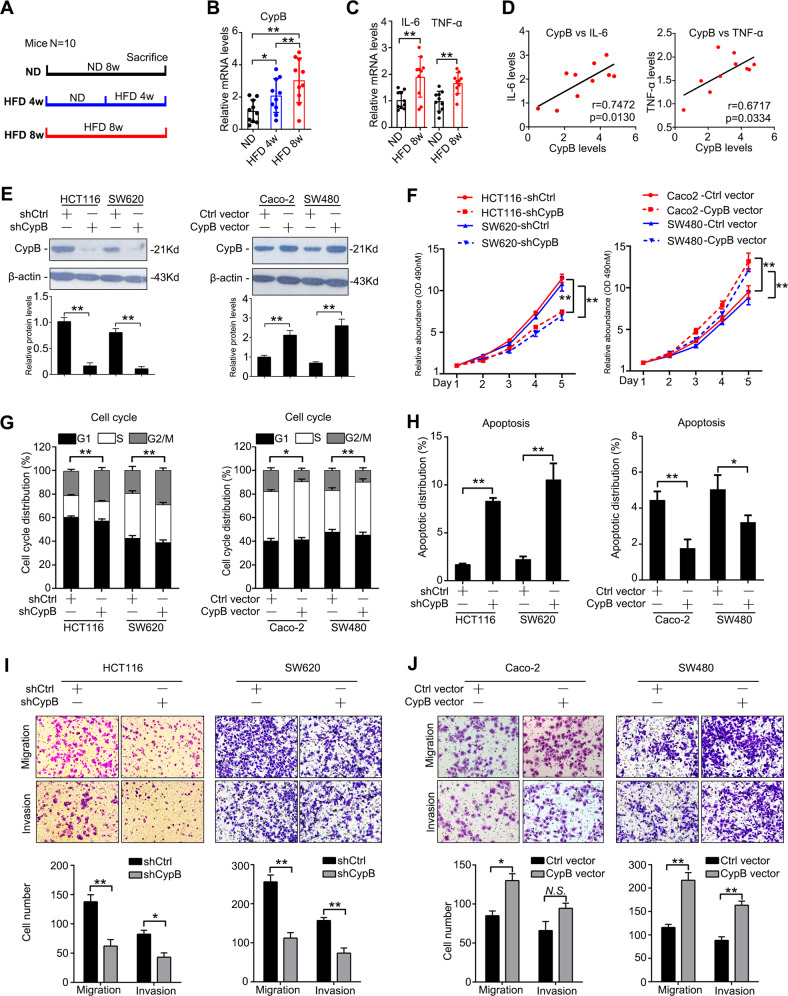
CypB increases CRC cell growth, invasion and metastasis in vitro. **A** Groups: C57BL/6 mice of 12 weeks old were treated with normal diet (ND), high-fat diet for 4 weeks (HFD 4w) or 8 weeks (HFD 8w) and sacrificed. *N* = 10 per group. **B**, **C** Colon tissues were collected and mRNA levels of CypB **B**, IL-6 and Tnf-α **C** were detected using qRT-PCR. β-actin were used as internal control. **D** Correlation of relative mRNA levels of CypB with that of IL-6 in colon (left) and correlation relative mRNA levels of CypB with that of Tnf-α (right). **E** Immunoblots showing CypB expression in HCT116 and SW620 cells infected with Lenti-shCypB or control, and Caco-2 and SW480 cells infected with CypB overexpressing vector. **F** Left: XTT assay of HCT116 and SW620 cells infected with Lenti-shCypB. *N* = 5. Right: XTT assay of Caco-2 and SW480 cells infected with CypB vector. *N* = 5. Cell cycle analysis **G** and apoptosis analysis **H** of Lenti-shCypB-infected HCT116 and SW620 cells (left) and CypB-vector infected Caco-2 and SW480 cells (right). Apoptosis was induced by serum-free medium for 24 h. Means ± SD of a representative experiment (*n* = 3) performed in triplicates are shown. **I** Representative images in transwell assays of migration and invasion using Lenti-shCypB-infected HCT116 and SW620 cells (top) and statistical analysis (bottom). **J** Representative images in transwell assays of migration and invasion using Lenti-CypB-infected HCT116 and SW620 cells (top) and statistical analysis (bottom). The data are presented as the means ± SDs. *P < 0.05; **P < 0.01; N.S. not significant.

To investigate whether CypB is involved in high-fat diet induced CRC growth regulation, C57BL/6 mice were treated with normal diet (ND) and high fat diet (HFD) (Fig. 2A). Overexpression of CypB were detected in colon tissues in HFD group (Fig. 2B). Similarly, proinflammatory cytokines such as IL-6 and TNF-α were also found to be induced by HFD (Fig. 2C). CypB expression were positively correlated with expression of IL-6 and TNF-α (Fig. 2D), suggesting a possible role of CypB in HFD-induced colon inflammation. To further determine function of CypB, HCT116 and SW620 cells were then infected with lentiviral vectors expressing shRNA against CypB or a control, while Caco-2 and SW480 cells were infected with CypB vectors and control vectors. The expression of CypB were evaluated by immunoblotting after transfection (Fig. 2E). XTT assays revealed that cell growth was significantly reduced by CypB downregulation compared with the control, while restoration of CypB increased cell growth (Fig. 2F). Moreover, cell cycle assays showed that silencing CypB increased the G2/M population and reduced the S and G0/G1 population compared with control cells, while CypB overexpression increased S phase population (Fig. 2G). Apoptosis assays further revealed that CypB inhibition led to an increased percentage of apoptotic CRC cells, whereas CypB overexpression decreased percentages of apoptosis (Fig. 2H). Furthermore, transwell assays showed that silencing CypB in HCT116 and SW620 cells induced decreased invasion and metastasis (Fig. 2I), while upregulation of CypB in Caco-2 and SW480 cells increased invasive and metastatic potential (Fig. 2J). These data showed that HFD-induced CypB increased CRC cell growth, invasion and metastasis in vitro.

### CypB increases CRC cell growth, invasion and metastasis in vivo

**Fig. 3:**
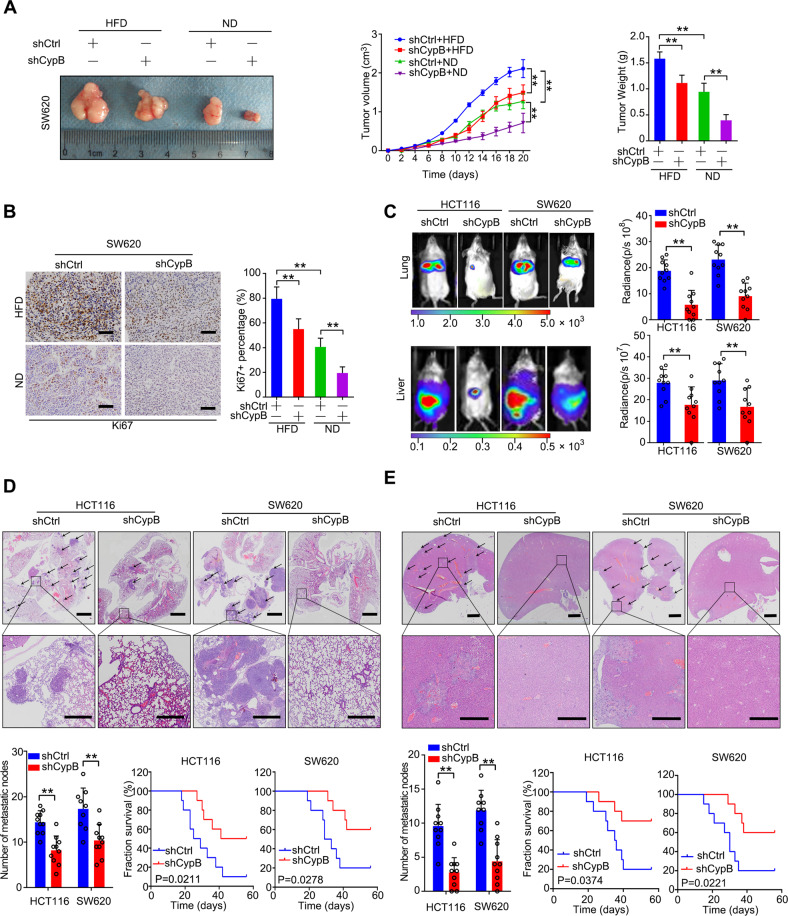
CypB increases CRC cell growth, invasion and metastasis in vivo. **A** Left: Representative images of tumors from SCID mice xenografted with SW620 cells infected with lentiviral shCypB or shNC vectors, with normal diet (ND) or high fat diet (HFD) (*N* = 5). Middle: Volume of xenografted tumors derived from SW620 cells. Right: tumors weight of different groups. **B** Left: Representative images of tumor samples that were stained via IHC for Ki-67 staining. Right, the percentages of Ki-67-positive cells. Scale bar: 100 μm. **C** Top: Representative bioluminescent images and radiance levels of lung metastasis in the different groups at 8 weeks after tail vein injection (*N* = 10). Bottom: Representative bioluminescent images and radiance levels of liver metastases in the different groups at 8 weeks after intrasplenic inoculation. **D** Representative haematoxylin and eosin (HE) staining showing metastatic nodules in lung tissues of SCID mice (*N* = 10). Scale bar: 2 mm (upper); 500 μm (lower). Arrows show metastatic nodes of tumors. Bottom: Left, number of metastatic nodes in different groups. Right, overall survival times of the SCID mice in the different groups. **E** Representative HE staining showing metastatic nodules in liver tissues of SCID mice (*N* = 10). Scale bar: 2 mm (upper); 500 μm (lower). Bottom: Left, number of metastatic nodes in different groups. Right, overall survival times of the SCID mice in different groups. The data are presented as the means ± SDs. *P < 0.05; **P < 0.01; N.S. not significant.

To extend the above findings to an in vivo setting, SW620 cells infected with CypB shRNA as Fig. 2A were subcutaneously injected into the flanks of SCID mice treated with ND or HFD. Analysis showed that HFD indeed promoted the growth of tumor, while silencing CypB in SW620 cells caused dramatic reductions in tumor weight and volume in mice (Fig. 3A). Ki-67 staining in xenografts also showed that CypB-downregulated cells exhibited decreased proliferation (Fig. 3B). To further determine the role of CypB in CRC metastasis, we established lung and liver metastasis models by injecting CypB-silenced HCT116 and SW620 cells into the tail veins and spleens of SCID mice, respectively. Compared to the control condition, silencing CypB decreased the incidence of lung metastasis (Fig. 3C up) and the number of metastatic nodules (Fig. 3D), and improved survival in mice (Fig. 3D). Similarly, CypB downregulation reduced liver metastasis following spleen injection (Fig. 3C down, Fig. 3E). These results indicate that silencing CypB suppress tumor progression and reinforce responsiveness to chemotherapy of CRC cells in vivo.

### LncRNA PVT1 directly interacts with CypB

**Fig. 4:**
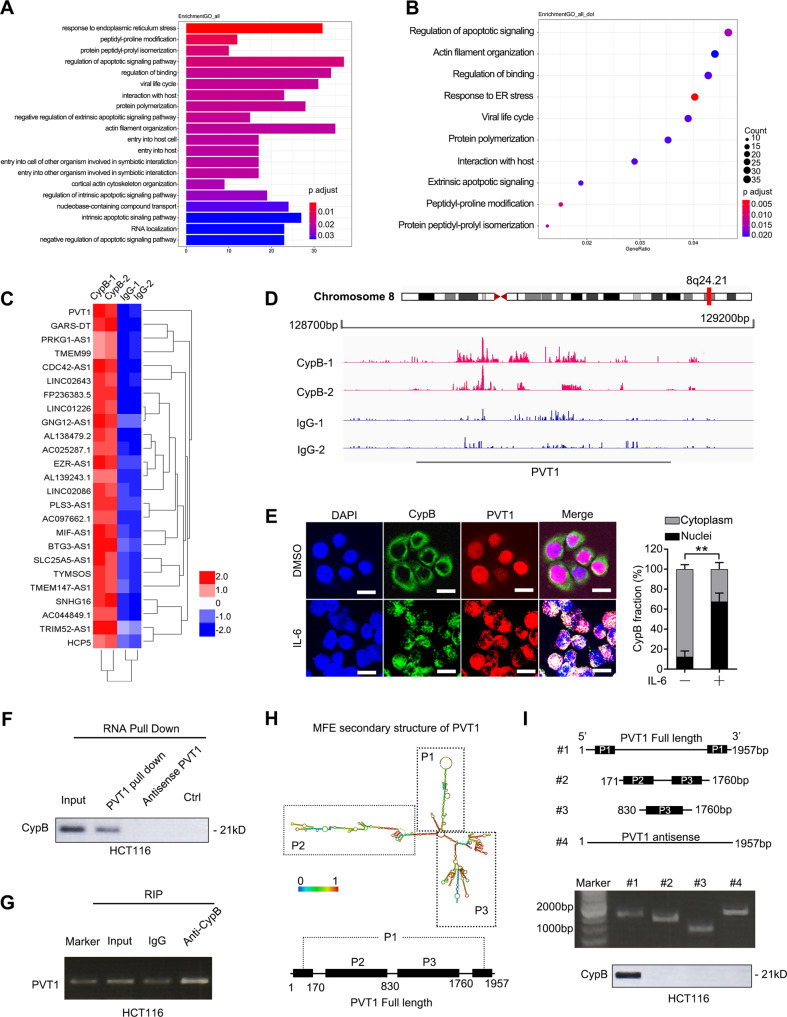
LncRNA PVT1 interacts with CypB. **A**, **B** GO analysis of CypB binding RNA using RIPseq. **C** Heatmap of 25 most enriched lncRNAs binding to CypB protein determined by RIP-seq. **D** Mapping of RIP-seq reads back to genomic locus of LncRNA-PVT1. **E** Left: Representative images of co-localization analysis of CypB and PVT1 in HCT116 cells treated with IL-6 (50 ng/mL) or DMSO for 30 min using protein IF and RNA FISH assays respectively. Scale bars: 20 μm. Right: Statistical analysis for percentages of cells with nuclear co-localization of CypB with lncPVT1. **F** RNA pulldown assay with PVT1, followed by Western blotting using the indicated antibodies. **G** RNA-binding protein immunoprecipitation (RIP) assay for CypB followed by agarose gel electrophoresis. **H** Graphic illustration of the predicted secondary structure and liner structure of PVT1 generated by ViennaRNA Database. **I** Top: Agarose gel electrophoresis analysis of full-length (#1), truncated or antisense (#2–4) PVT1 transcripts; Bottom: immunoblots for CypB pulled-down by the indicated transcripts of PVT1. Representative of three independent experiments. The data are presented as the means ± SDs. *P < 0.05; **P < 0.01; N.S. not significant.

As emerging evidence has implicated that lncRNAs play vital roles in colon carcinogenesis [26], we set out to determine the functional connection between oncogene CypB and lncRNAs in CRC. RNA sequencing following RNA Binding Protein Immunoprecipitation (RIP) was performed using CypB antibody and total cell RNA of HCT116 cells treated with IL-6, which showed that multiple lncRNAs were captured by CypB (Supplementary Fig. S2A). GO analysis showed that CypB-binding RNAs were enriched in pathways of endoplasmic reticulum stress, apoptotic signaling and actin filament organization (Fig. 4A, B, Supplementary Fig. S2B–D), which are essential in tumor growth, survival and metastasis [27], indicating that CypB-binding lncRNAs may be involved in CypB’s oncogenic roles in CRC. The 20 most abundant lncRNAs binding to CypB were shown in the heatmap (Fig. 4C) and further validated by RIP-qPCR analysis (Fig. S3A). Compared to IgG, several candidate lncRNAs were screened and confirmed to bind CypB appreciably with p value < 0.05 (Supplementary Fig. S3A, B). Among them, lncRNA PVT1 was chosen for further investigation not only because it was reported to be an oncogene in various cancer types[28], but also because it was found to be an important regulator in STAT3 function in cancer cells [29] and we previously showed that CypB is also essential for STAT3 activation [14].

Further evidence demonstrated that PVT1 binds to CypB. First, the coverage tracks from the RIP-seq showed that the CypB-bound RNAs cover genomic position of PVT1 (chromosome 8q24.21, nucleotides 127,794,533-128,101,253) (Fig. 4D). Besides, the assays combining RNA FISH with protein immunofluorescence showed that CypB, which translocate from cytoplasm to nucleus, is strongly co-localized with PVT1 in HCT116 cells upon IL-6 treatment (Fig. 4E). Moreover, CypB was immunoblotted in RNA pull-down products using biotin-labeled oligo probe sets targeting PVT1 (Fig. 4F). Meanwhile, this was validated by RIP assays which showed that PVT1 was significantly enriched in pull-downs using antibodies against CypB compared to control IgG (Fig. 4G). To determine the specific region of PVT1 that binds to CypB, a series of PVT1 mRNA fragments were generated based on its secondary structure predicted by ViennaRNA Database (http://rna.tbi.univie.ac.at/) (Fig. 4H) and then these constructs were used in biotin-labeled RNA pull-down assays. The results showed that the PF00160 domain of CypB mainly binds to a PVT1 fragment that is transcribed from either nucleotide 1 to 170 or 1760 to 1957 (Fig. 4I, Supplementary Fig. S3C). Together, these results demonstrated that LncRNA PVT1 directly interacts with CypB in CRC cells.

### LncRNA PVT1 promotes CRC growth and metastasis

**Fig. 5:**
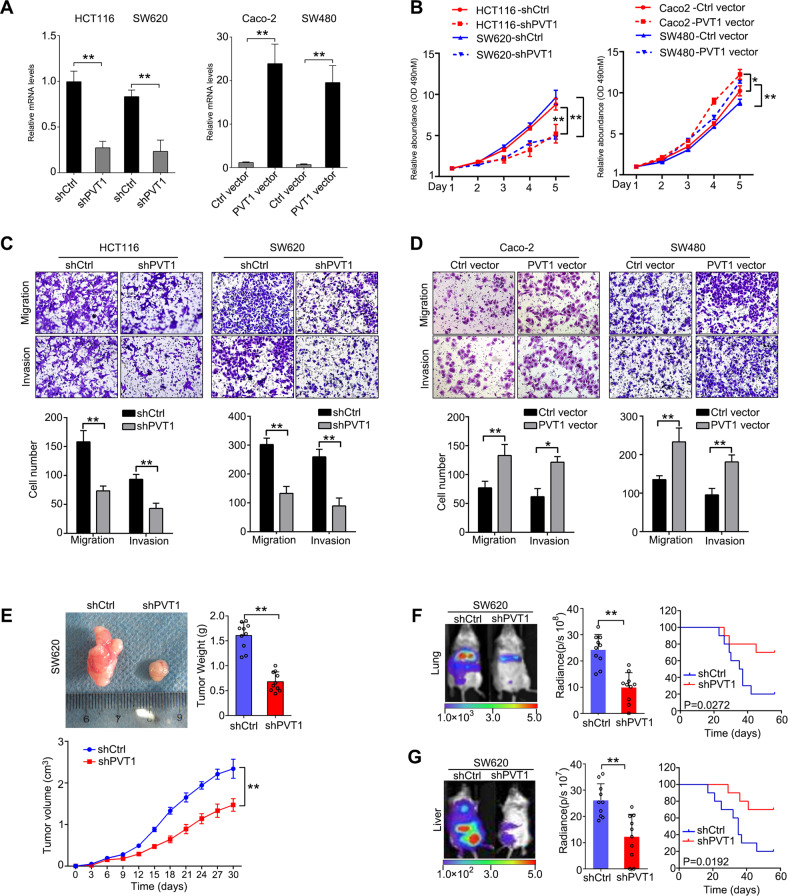
LncRNA PVT1 promotes CRC growth and metastasis. **A** Left: qRT-PCR assays showing PVT1 expression in HCT116 and SW620 cells infected with Lenti-shPVT1 or control (left), and Caco-2 and SW480 cells infected with PVT1 overexpressing vector (right). **B** Left: XTT assay of HCT116 and SW620 cells infected with Lenti-shPVT1 (*N* = 5). Right: XTT assay of Caco2 and SW480 cells infected with PVT1 vector (*N* = 5). **C** Representative images in transwell assays of migration and invasion using Lenti-shCypB-infected HCT116 and SW620 cells (top) and statistical analysis (bottom). **D** Representative images in transwell assays of migration and invasion using Lenti-CypB-infected HCT116 and SW620 cells (top) and statistical analysis (bottom). **E** Representative images of tumors from SCID mice subcutaneously xenografted with SW620 cells infected with lentiviral shPVT1 or shNC vectors (top left, *N* = 5). Tumor weight (top right) and volume (bottom) of xenografted tumors derived from SW620 cells. **F** Left and Middle: Representative bioluminescent images and radiance levels of lungs in the different groups at 8 weeks after tail vein injection (*N* = 10). Right: Overall survival times of the mice in the different groups. **G** Left and Middle: Representative bioluminescent images and radiance levels of livers in the different groups at 8 weeks after spleen injection (*N* = 10). Right: Overall survival times of the mice in the different groups. The data are presented as the means ± SDs. *P < 0.05; **P < 0.01; N.S. not significant.

To investigate the function of PVT1 with regards to CRC cell growth, HCT116 and SW620 cells were infected with PVT1 shRNAs, while Caco-2 and SW480 cells were infected with overexpressing vectors, and PVT1 expression was confirmed by qRT-PCR (Fig. 5A). Cell proliferation assays indicated that PVT1 shRNA significantly inhibited CRC cell growth, while restoration of PVT1 increased CRC cell proliferation (Fig. 5B). Cell cycle analysis showed that PVT1 shRNA induced G2/M arrest, whereas PVT1 overexpression reduced the proportion of cells in G2/M (Supplementary Fig. S4A). Furthermore, PVT1 shRNA increased the proportion of cells undergoing apoptosis, while PVT1 restoration reduced the number of apoptotic cells (Supplementary Fig. S4B). Besides, PVT1 shRNA also decreased tumor cell invasion and metastasis in HCT116 cells and SW620 cells (Fig. 5C), while PVT1 upregulation increased these phenotypes in Caco-2 and SW480 cells (Fig. 5D). The effects of PVT1 on CRC progression were also studied in vivo: SW620 cells infected with lentiviral vectors expressing PVT1 shRNA were subcutaneously injected into the right flanks of SCID mice. At 30 days post-injection, the mean xenograft tumor volume and weight was significantly lower for shPVT1-SW620 xenografts than for control xenografts (Fig. 5E). Lung and liver metastasis models using SW620 cells further showed that silencing PVT1 decreased the incidence of lung and liver metastasis, and improved survival in mice (Fig. 5F, G). These results provided evidence showing LncRNA PVT1 promotes CRC cell growth and metastasis in vitro and in vivo.

### PVT1 promotes CRC cell growth through CypB-STAT3 axis

**Fig. 6:**
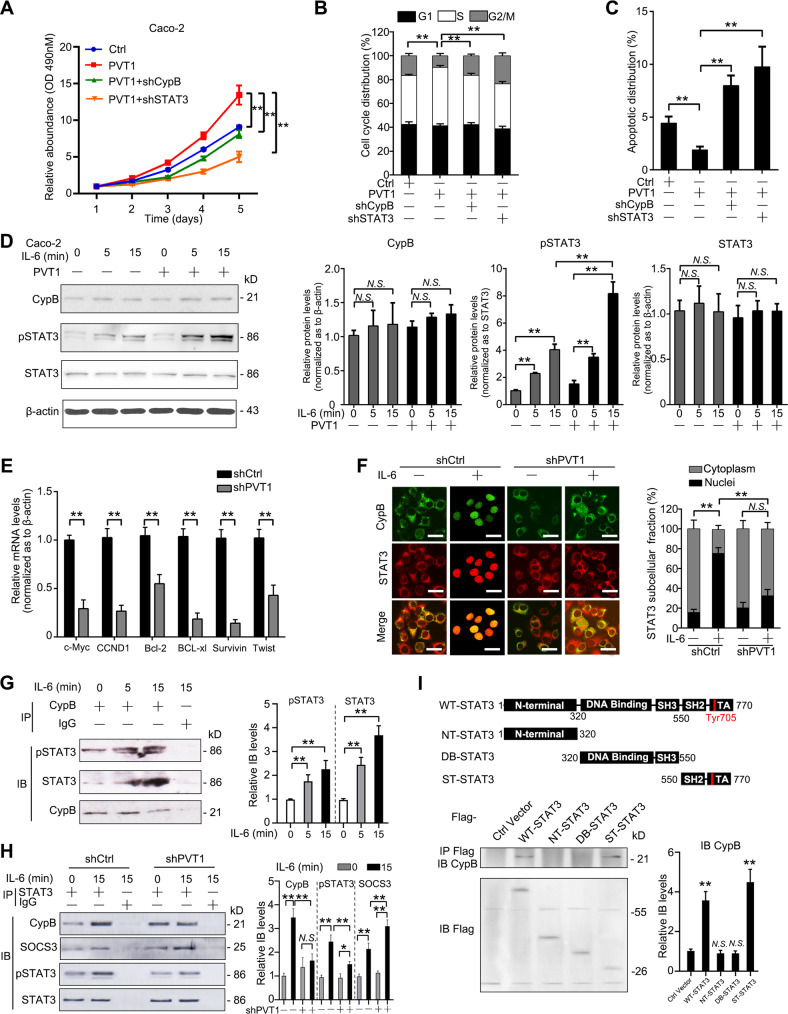
PVT1 promotes CRC cell growth through CypB-STAT3 axis. **A** XTT assay of Caco-2 cells infected with Lenti-shPVT1, co-infected with either shCypB or STAT3 vectors and corresponding negative control as in Fig. S5A. Cell cycle analysis **B** and apoptosis analysis **C** using FACS in Caco-2 cells. **D** Expression of CypB, STAT3, and phosphorated STAT3 by immunoblots in Caco-2 cells upon IL-6 treatment (50 ng/mL) for 0–15 min. **E** qRT-PCR results of STAT3 downstream targets including c-Myc, CCND1, Bcl-2, Bcl-xl, Survivin, Twist, in Caco-2 cells infected with PVT1 shRNAs. **F** Representative images of IF analysis of CypB (green) and STAT3 (red) in Caco-2 cells infected with PVT1 shRNAs or control while being treated with IL-6 (50 ng/mL) for 15 min. Scale bars: 20 μm. Representative images of three independent experiments were shown. Right: Quantitation of STAT3 distribution. **G, H** Reciprocal co-IP assay showing interaction of CypB with endogenous STAT3 and phosphorated STAT3 in IL-6-treated Caco-2 cells, using beads coated with CypB antibody **G** and STAT3 antibody **H** or respective IgG. Bands were quantified and normalized as to CypB levels in **G** and STAT3, pSTAT3(Tyr705) and SOCS3 in **H**. Representative images of three independent experiments were shown. **I** Tagged STAT3 and truncates were constructed and transfected into Caco2 cells. After IL-6 treatment for 15 min, cell lysates were collected, precipitated with Flag antibody and immunoblotted with CypB antibody. The data are presented as the means ± SDs. *P < 0.05; **P < 0.01; N.S. not significant.

We previously reported that STAT3 activation upon IL-6 treatment was exaggerated by CypB and their interaction in nuclei activate transcription of downstream genes [14]. Combining these results and the findings suggesting direct interaction of PVT1 and CypB, we were interested in whether the connection between PVT1 and CypB play roles in STAT3 function in cancer cells. We used STAT3 shRNA and CypB shRNA in Caco-2 cells transfected with PVT1 overexpressing vectors. Expression of CypB and phosphorylation of STAT3 were validated by immunoblotting, showing that phosphorylation of STAT3 at Tyr705 were inhibited by knockdown of either CypB or STAT3(Supplementary Fig. S5A). Proliferation assays showed that silencing either CypB or STAT3 rescued the promotion of growth induced by PVT1 restoration (Fig. 6A). Besides, CypB shRNA or STAT3 shRNA also abrogated the increase of S-phase cell distribution induced by PVT1 upregulation (Fig.6B), as well as the PVT1-caused decrease of apoptotic population (Fig. 6C). Moreover, transwell assays showed similar results, suggesting that knockdown of either CypB or STAT3 decreased PVT1-induced migration and invasion (Supplementary Fig. S5B). These data suggest CypB-STAT3 axis may mediate the PVT1 function on tumor growth and metastasis.

Caco-2 cells were then treated with IL-6 for 0–15 min followed by transfection of PVT1 vectors. Immunoblotting results showed that the IL-6-induced time-dependent increase of STAT3 phosphorylation (Tyr705) was also promoted by the restoration of PVT1 (Fig. 6D). Meanwhile, silencing PVT1 induced decrease of transcription of STAT3 downstream targets including c-Myc, CCND1, Bcl-2, BCL-xL, Survivin and Twist (Fig. 6E), which were considered to play vital roles in the regulation of cell cycle, apoptosis, and metastasis. More interestingly, confocal immunofluorescence showed that CypB co-localized with STAT3 in the nucleus following IL-6 treatment as expected, while upon PVT1 knockdown using shRNA, the number of cells with the nuclear distribution of STAT3 was significantly decreased (Fig. 6F). Meanwhile, immunoblotting assays showed that upon IL-6 stimulation for 30 min, the nuclear protein levels of STAT3 and CypB were both increased, while knockdown of lncPVT1 partially abrogated the elevation of nuclear protein levels of CypB and STAT3 (Supplementary Fig. S6A–C). We then evaluated whether CypB directly associates with STAT3. Indeed, using co-immunoprecipitation (co-IP) assays, CypB was found to interact with STAT3, which was enhanced by IL-6 stimulation (Fig. 6G). Interestingly, the CypB-STAT3 interaction upon IL-6 treatment was suppressed by PVT1 knockdown, while SOCS3-STAT3 interaction were increased (Fig. 6H). Finally, Flag-tagged STAT3 vectors and truncated vectors were constructed and transfected into Caco2 cells. Co-IP results showed that CypB bind the SH2-TA domain of STAT3, containing the phosphorylation and activation site Tyr705 (Fig. 6I), possibly explaining the reason why CypB exaggerated IL-6-triggered STAT3 activation. Taken together, these results suggest that PVT1 promoted CRC cell growth and metastasis through activating interaction and nuclear translocation of CypB-STAT3 complexes.

### STAT3 Promotes PVT1 transcription by directly binding to its promoter

**Fig. 7:**
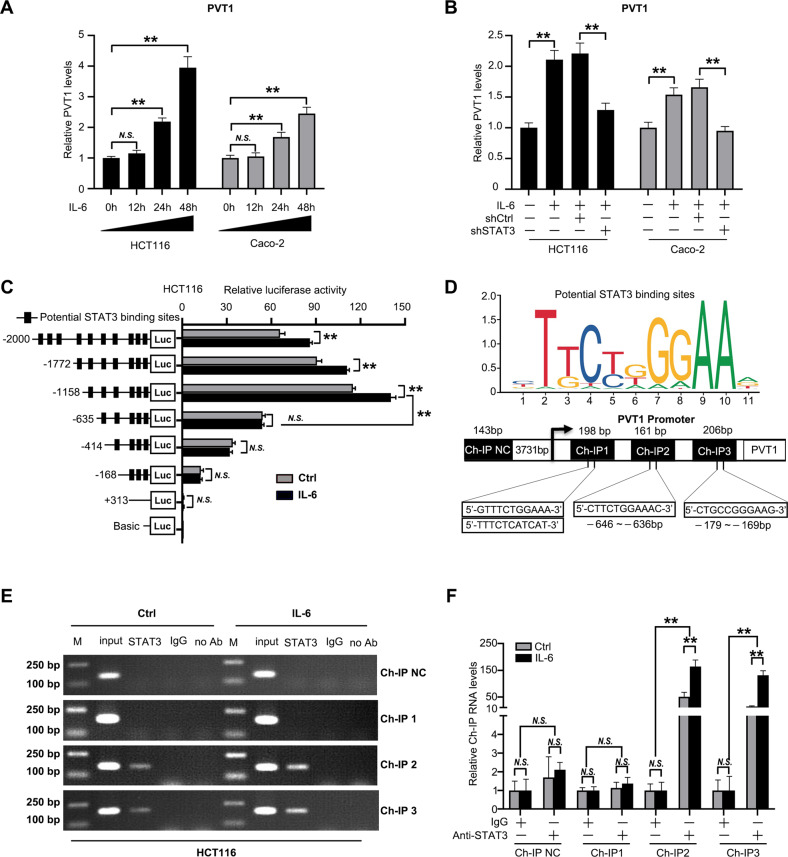
STAT3 Promotes PVT1 transcription by directly interacting with its promoter. **A** HCT116 and Caco-2 cells were treated with IL-6 at concentration of 50 ng/mL for 0–48 h and expression of PVT1 were examined using qRT-PCR. GAPDH were used as internal control. **B** HCT116 and Caco-2 cells were infected with STAT3 shRNA and treated with IL-6 (50 ng/mL) or control for 24 h and expression of PVT1 were examined using qRT-PCR. **C** Serially truncated PVT1 promoter constructs were cloned to pGL3-luciferase reporter plasmids and transfected into HCT116 cells. 48 h after transfection, the relative luciferase activities were determined after IL-6 (50 ng/mL) treatment for 30 min. **D** Top: Schematic diagram showing the binding motifs of STAT3 in the PVT1 promoter. Bottom: Ch-IP targets the promoter of PVT1. **E** A Ch-IP assay demonstrated the direct binding of STAT3 to the PVT1 promoter in HCT116 cells. M: Marker. **F** qRT-PCR of the Ch-IP products validated the binding capacity of STAT3 to the PVT1 promoter. The data are presented as the means ± SDs. *P < 0.05; **P < 0.01; N.S. not significant.

To further explore the mechanism by which PVT1 is upregulated in CRC cells, we analyzed potential TF binding motifs in the promoter region of PVT1 in JASPAR database. Interestingly, 13 STAT3 binding sites were identified in PVT1 promoter (Supplementary Table S4), suggesting that PVT1 may be a potential downstream transcript of STAT3. HCT116 cells and Caco2 cells were then treated with IL-6. Interestingly, PVT1 expression was induced in a time-dependent manner (Fig. 7A), which can be blocked by STAT3 knockdown (Fig. 7B), strongly indicating that STAT3 positively regulates PVT1 expression. Thus, we tested if STAT3 directly targets PVT1. We generated a series of PVT1 promoter truncation mutants and determined whether STAT3 transcriptionally promotes PVT1. A luciferase assay after IL-6 treatment showed that the regulatory region might be between −1158 and −414 bp (Fig. 7C). Site-directed mutagenesis of the PVT1 promoter were generated (Supplementary Fig. S6A), which further showed that both STAT3-binding sites (−646 to −636bp and −179 to −169bp) are the predominant sites for STAT3-mediated transcriptional activation (Fig. 7D, Supplementary Fig. S6B). Chromatin immunoprecipitation assay (ChIP) assays further confirmed that STAT3 binds to the two sites of PVT1 promoter in HCT116 cells (Fig. 7E). Consistently, qRT-PCR of ChIP products showed that IL-6 treatment significantly increased the association of STAT3 with the PVT promoter (Fig. 7F). Together, these results indicate that the IL-6/STAT3 pathway directly promotes PVT1 transcription in CRC cells.

### The PVT1/CypB/STAT3 pathway is characteristic of primary CRC tissues

**Fig. 8:**
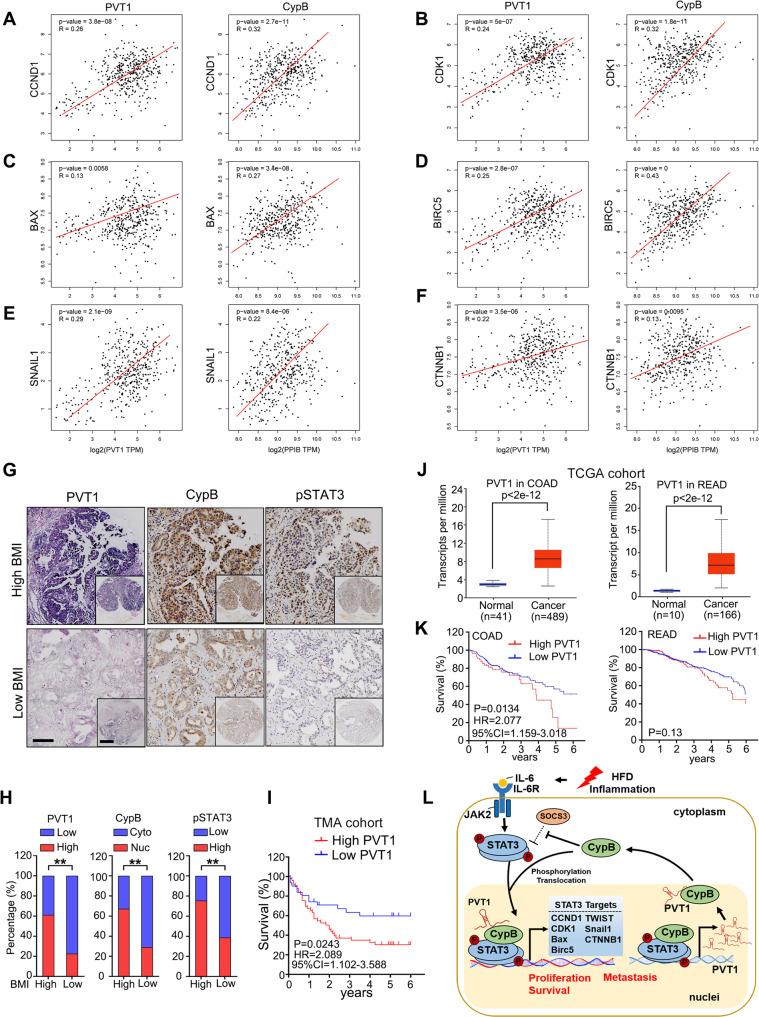
The PVT1/CypB/STAT3 pathway is characteristic of primary CRC tissues. **A**–**F** Correlation of PVT1 and CypB with STAT3-downstream targets using TCGA data of COAD and READ by GEPIA database (http://gepia.cancer-pku.cn/). Y-axis, transcripts per million (TPM) of STAT3 targes; X-axis, TPM of PVT1 and CypB. Expression of PVT1 and CypB were positively correlated with CCND1 **A**, CDK1 **B**, BAX **C**, BIRC5 **D**, SNAIL1 **E**, and CTNNB1 **F**. **G** The cases in TMA cohort were divided into two groups: high BMI (BMI ≥ 26) and low BMI (BMI <26). The representative images of high level of PVT1, nuclear CypB and pSTAT3 were shown in upper panel, while the low level of PVT1, nuclear CypB and pSTAT3 were shown lower panels. Scale Bars: 100 μm (main); 400 μm (inset). **H** The levels of BMI levels were positively correlated with expression of PVT1, CypB and pSTAT3 in CRC tissues. **I** Kaplan-Meier curves of patients with low vs high expression of PVT1 in TMA cohort. **J** PVT1 levels in TCGA data of COAD (left, *P* < 2e-12) and READ (right, *P* < 2e-12). **K** Kaplan-Meier curves of patients with low vs high expression of PVT1 in TCGA cohort of COAD (left, *n* = 286; *P* = 0.0134, HR = 2.077) and READ (right, *P* = 0.13). **L** Proposed working model in this study showing HFD-induced IL-6/STAT3 activation expedited by CypB, followed by STAT3 nuclear translocation and transcription of STAT3 targets, which increase cell proliferation, survival, and metastasis. A feedforward regulation of STAT3 promotes PVT1 transcription and its interaction with CypB, which reciprocally promotes STAT3 activation.

Finally, to test whether the regulation described above in CRC cell lines is clinically relevant, TCGA cohorts focusing on PVT1 and CypB were studied. Correlation analysis showed that both PVT1 expression and CypB expression were both positively correlated with STAT3 targets in cell cycle including CCND1 (Fig. 8A left: R = 0.26, *P* = 3e^−8^; Right: R = 0.32, *P* = 2.7e^−11^) and CDK1 (Fig. 8B left: R = 0.24, *P* = 5e^−7^; Right: R = 0.32, *P* = 1.8e^−11^). Besides, apoptosis-related regulators regulated by STAT3 such as Bax (Fig. 8C left: R = 0.13, *P* = 0.0058; Right: R = 0.27, *P* = 3.4e^−8^) and BIRC5 (Fig. 8D left: R = 0.25, *P* = 2.8e^−7^; Right: R = 0.43, *P* = 0) were also found to be positively related with PVT1and CypB. Moreover, similar results were also found in correlation among PVT1, CypB and STAT3 downstream SNAIL1, CTNNB1, which play pivotal roles in metastasis (Fig. 8E left: R = 0.29, *P* = 2.1e^−9^; Right: R = 0.22, *P* = 8.5e^−6^; Fig. 8F left: R = 0.22, *P* = 0.3.5e^−6^; Right: R = 0.137, *P* = 0.0095). These results were consistent with GSEA assays on CypB in TCGA data (Fig. 1F) and GO analysis of RIPseq results (Fig. 4A, B). Similar results were also found in STAT3-associated interleukins such as IL-1B, IL-6, IL-11, CCL3, CXCL8, CXCL10, LIF (leukemia inhibitory factor) and OSM (oncostatin M) using COAD and READ data in TCGA cohorts (Supplementary Fig. S7A–H). These clinical data further indicate cooperative effects of CypB and PVT1 in promoting tumorigenesis during inflammation, which is triggered by HFD and maintained by constitutive activation of STAT3 pathway.

We also measured PVT1 expression by in situ hybridization (ISH) in the TMA cohort. Compared with normal tissues, PVT1 levels were increased in CRC tissues (Fig. 8G). In addition, we found that patients with higher BMI tended to have increased PVT1 levels, nuclear expression of CypB and pSTAT3 expression (Fig. 8G). The 80 CRC patient cases were then divided into groups with relatively high or low levels of BMI, PVT1, CypB, and pSTAT3. From this analysis, we observed a positive relation of BMI with PVT1, nuclear CypB and pSTAT3 expression (Fig. 8H). We also found that patients with high PVT1 expression had significantly poorer prognoses than those with low PVT1 expression (Fig. 8I). Further expression analysis on TCGA data showed that PVT1 transcripts were extremely higher in cancer tissues of COAD and READ, compared with that in normal tissues (Fig. 8J). Kaplan–Meier analysis further revealed that high-level PVT1 expression was associated with shorter disease-free survival time for COAD patients, but not for READ patients (Fig. 8K). In Summary, these results showed that the inflammatory PVT1/CypB/STAT3 axis is active in primary human colorectal carcinogenesis.

## Discussion

Previous studies suggest that elevated CypB expression significantly contributes to the survival and proliferation of cancer cells in several kinds of solid tumor [13, 20, 30]. But few study have elucidated the function of CypB in terms of inner links between HFD-associated inflammation and CRC. In this study, we investigated roles of HFD-induced CypB in regulation of proliferation and metastasis of CRC, and identified an IL-6-triggered circuit involving STAT3-mediated transcription of PVT1 and its binding-partner CypB which facilitates STAT3 activation, connecting chronic inflammation with colorectal carcinogenesis and metastasis.

CypB is mainly located in ER and attenuates ER stress-induced cell injury by interacting with the ER-related chaperones [19], which is associated with the malignant progression and regulation of a variety of tumors [12–14, 19, 20]. It is previously reported to promote gastric carcinogenesis and inflammation-cancer progression through the activation of STAT3 in our recent study [14]. But its research in CRC is rarely reported. Herein, our data based on TCGA cohorts and independent cohort of tissue microarrays showed that levels of CypB transcripts and protein are both increased in CRC tissues, indicating its potential role in promoting the progression of CRC. This is consistent with our previous findings in stomach cancer [14] and other kinds of solid tumors [12, 13, 19]. The mechanism of upstream regulation of CypB were explored in several recent studies including our previous work, showing that hypoxia-associated Activating transcription factor 4 (ATF4) transcription and IL-6 induced post-transcriptional regulators such as microRNA were both responsible for upregulation of CypB in cancer [20, 31]. Another results that should be noted is that serum CypB levels of CRC patients were tested as CypB can be secreted into blood and results showed that serum CypB detection may be a novel, non-invasive approach for early CRC screening. One recent study reported that 27-hydroxycholesterol induced inflammatory pathways including the phosphorylation of NF-κB p65 accounted for the overexpression of CypB [22]. Similarly in this study, we found that CypB in sera of CRC patients is positively associated with BMI of patients. Combining these clinical data with in vivo results showing CypB expression in colon tissues was promoted by HFD, which is associated with proinflammatory cytokines, we suggest that HFD-induced CypB may be involved in increased proliferation and metastasis in CRC.

Recent studies indicated that CypB promotes proliferation and survival of cancer cells [12, 13, 16, 19, 20], while few studies have focused on its effects on metastasis. Thus, we further tested if CypB promotes tumor cell growth and metastasis by establishing gain-of- and loss-of-function models using lentiviral vectors. Indeed, knockdown of CypB suppressed HFD-supported tumor cell growth by inducing G0/G1 cell cycle arrest and apoptosis. Meanwhile, CypB downregulation also decreased cell migration and invasion in vitro and inhibited lung and liver metastasis in immunodeficiency mice. It is proved in many studies that CypB protected tumor cells from stress-induced apoptosis mostly through activation of STAT3 signaling pathway [14, 15, 30, 32]. Similarly in this study, co-localization of CypB with STAT3 in nuclei of cell were detected in CRC cell lines in response to IL-6 treatment, which is typical stimulator for activation of STAT3. We further found that silencing STAT3 rescued CypB- induced proliferation, migration and metastasis. Direct interaction of STAT3 and CypB was found in CRC cell lines, suggesting a possible mechanism of protein modification on STAT3 protein by CypB. This is consistent with our previous findings and other studies [12, 14, 30] showing CypB’s important effects in STAT3 activation and transcription of its downstream targets which regulates cell proliferation and survival. Tumorigenesis of CRC is involved with the transformation of normal colorectal epithelium to an invasive and metastatic tumor in response to chronic stimulation of inflammation, which is considered to be triggered by STAT3 [9]. Thus, our study may provide new appealing anti-cancer strategy to inhibit the oncogenic functions of STAT3 by targeting CypB in CRC.

lncRNAs often function through binding to chaperone proteins [33, 34]. As a typical chaperone protein coordinates with ER-stress and STAT3 activation, CypB were found to be translocated into cell nuclei together with STAT3 following IL-6 treatment [12, 14]. We explored if CypB interact with functional RNAs in nuclei. Indeed, our RIPseq results here showed that CypB directly bound to lots of lncRNAs, which were enriched in signaling pathways including regulation of ER-stress, peptidyl-prolyl cis/trans isomerase (PPIase) and apoptosis, suggesting these ncRNAs may be functional as CypB itself were proved to participate in these processes [16, 18, 19]. After screening by integrating the sequencing results and online database, LncRNA PVT1 were chosen for further study [28, 35, 36], not only because it has been shown to have important oncogenic features in several types of cancer, but also because it was reported to interact directly with STAT3 in cancer cells [29]. LncRNA PVT1 transcription site is located in a cancer susceptibility locus downstream of Myc, and ablation of PVT1 from MYC-driven colon cancer line HCT116 diminished its tumorigenic potency [37]. Herein, we found that PVT1 physically binds to CypB domain (PF00160), which was detected from RIPseq results and validated by RIP and RNA pull-down technology. We further showed that knockdown of PVT1 suppressed tumor cell proliferation and metastasis in vitro and in vivo, while ectopic expression of PVT1 increased tumor growth and migration, which were rescued by silencing of either CypB or STAT3. Besides, our results showing nuclear translocation of CypB and following co-localization with lncPVT1 in response to IL-6 treatment indicate potential roles of lncPVT1 and CypB in mediating activation of STAT3 in colon cancer cells. However, future studies will be needed to elucidate the specific effects of PVT1 on CypB/STAT3 complex assembling and nuclear translocation.

More interestingly, silencing PVT1 impaired IL-6 triggered STAT3 activation and decreased transcription of downstream targets of STAT3 by inhibiting its interaction with CypB and following phosphorylation and nuclear translocation. This may be due to its dual effects of sponging CypB and STAT3, which were proved in this study and a recent study [29], respectively. To date, most studies focusing on PVT1 reported its oncogenic function were exerted mainly through sponging tumor suppressive microRNAs [38]. For example, PVT1 were found to be upregulated in pancreatic cancer and overcome gemcitabine resistance through sponging miR-619- 5p, leading to upregulation of Pygo2, which is a downstream target of miR-619-5p [39]. However, our study demonstrated that PVT1 regulates CRC carcinogenesis and metastasis through directly binding to the two sequential signaling factors of CypB/STAT3 pathway, which were also proved binding to each other. This is particularly interesting because it is one of the few studies indicating PVT1 exert its action through binding to oncogenic proteins, instead of by sponging oncosuppressor miRNAs. The binding of PVT1 with CypB did not seem to affect the protein levels, while stabilizing the interaction between CypB and STAT3 and promoting the nuclear translocation of the CypB/STAT3 complex in CRC cells treated with IL-6. Besides, binding of CypB with SH2-TA domain of STAT3 were verified by Co-IP, containing the phosphor-site of Tyr705. This binding ameliorated the binding of STAT3 with SOCS3, a natural inhibitor of activated STAT3 dimer, indicating a possible mechanism of STAT3 activation caused by CypB. Whether protein modification of STAT3 and CypB is involved in the underlying mechanism will be explored in future study, since PVT1 were previously reported to stabilize target proteins through ubiquitination or acetylation [29, 40].

ncRNAs such as PVT1 and its targeted transcription factors (TF) often form feedforward loop in cancer progression [28], as ncRNAs themselves may be downstream transcripts of TF. Here we found that PVT1, supporting STAT3 activation by binding to CypB, was a transcriptional target of STAT3, forming a feedforward circuit in CRC cells. Recent studies have identified several ncRNAs that can be transcriptionally promoted or repressed by STAT3 [41–44], indicating a potential role for STAT3 in regulating a ncRNA network. STAT3 are important regulators of c-Myc, through binding to its promoter that is overlapping with E2F [45–47]. Given the fact that the PVT1 gene is located on 8q24.21 region that is close to Myc, it’s extremely interesting to determine if STAT3 regulate both Myc and PVT1 transcription by binding to their mutual promoter. Indeed, our results demonstrated that PVT1 expression was induced in a time-dependant manner following IL-6 treatment, which was abrogated by knockdown of STAT3. We predicted multiple potential binding sites within the PVT1 promoter. Further luciferase reporter assay and ChIP assay both showed that STAT3 directly binds to the two sites in PVT1 promoter including −646 to −636 bp and −179 to −169bp to CDS region. This demonstrates a new proof in inflammation-cancer transition of CRC involving CypB-supported STAT3 activation and their interaction with downstream ncRNAs induced by IL-6 cascades.

The integrative analysis of TCGA data and TMA cohort also support the results. In CRC tissues, we verified the positive correlation between PVT1/CypB and STAT3 phosphorylation. We showed that both PVT1 and CypB were significantly correlated with multiple STAT3 downstream targets, which are important regulators of cell cycle, apoptosis and metastasis. Meanwhile, expression of interleukins that are involved in STAT3 pathway, including IL-1, IL-6, IL-11, CCL3, CXCL8 etc, were also positively correlated with transcripts of CypB and PVT1 in TCGA cohorts of COAD and READ. These results suggest that the CypB/STAT3/PVT1 feedback loop is also present in clinical samples. STAT3 inhibition using Jak inhibitors such as Ruxolitinib and Momelotinib, has been practised in clinical treatment against metastatic CRC [48]. Recent studies also support that targeting specific oncogenic lncRNAs by ASOs have significant therapeutic value [49]. Thus, our study may be beneficial in STAT3-inhibitory therapy against metastatic CRC by developing ASO targeting PVT1.

## Conclusions

In summary, we elucidated the schematic model of CRC development shown in Fig. 8L. This figure depicts that HFD and following inflammatory IL-6 activates the STAT3 pathway and transcriptionally increase PVT1 expression, which binds to CypB and then aids STAT3 phosphorylation and nuclear translocation, resulting in STAT3 activation and increased CRC growth and metastasis. In this view, PVT1 and CypB may be important mediator that connects obesity and inflammation with constitutively activation of STAT3 in CRC cells and this new CypB/STAT3/PVT1 feedback loop may contribute to an improved understanding of inflammatory signaling in colorectal carcinogenesis during obesity.

## Materials and methods

### Tissue, serum, and cell lines

CRC tissue microarrays containing 80 cases of matched primary CRC tissues, metastatic lymph nodes, and adjacent non-cancerous tissue was purchased from Shanghai Outdo Biotech. Blood samples from 60 CRC patients (without overlap with the cases of tissue array) and 20 healthy volunteers were collected from Xi’an Central Hospital, Xi’an Jiaotong University, Xi’an, China. This study was approved by the Hospital’s Protection of Human Subjects Committee. 8 CRC cell lines (DLD-1, RKO, LoVo, HT-29, HCT-116, SW480, SW620, and Caco-2 cells) and immortalized human normal intestinal epithelial cells (HIEC cells) were used in this study. All cell lines were purchased from American Type Culture Collection (ATCC, Virginia, USA) and cultured in DMEM (Gibco, USA) with 10% fetal bovine serum (Gibco, USA). All cell lines were confirmed to be free of mycoplasma contamination.

### TCGA and GenomicScape data analysis

TCGA data were collected from the database of The Cancer Genome Atlas Program following the procedure as described [50] and CRC patients with a follow-up time exceeding 2000 days were excluded. A cohort [25] on GenomicScape database (http://www.genomicscape.com) were recruited to analyze the survival of CRC patients and results were generated online (Smith, Colon cancer 1 (Moffitt)). Gene set enrichment analysis (GSEA) was performed using GSEA software v2.07. Respective gene expression data and clinical information were further analyzed on GEPIA database (http://gepia.cancer-pku.cn/), UALCAN database (http://ualcan.path.uab. edu/), and LinkedOmics (http://www.linkedomics.org) [51].

### ELISA

Plasma levels of CypB were measured with an ELISA kit (R&D Systems) and CypB antibodies (Abcam) according to the manufacturer’s protocol as described before [14].

### Protein extraction and immunoblotting

Total proteins were prepared from cultured cell samples by complete cell lysis (Roche, Mannheim, Germany) with protease and phosphatase inhibitors. Nuclear proteins were prepared by Nuclear Protein Extraction Kit (Solarbio, Beijing, China) following the instruction from the manufacturer. Denatured proteins were separated on SDS-PAGE and transferred to membranes, followed by immunoblotting using antibodies of CypB (Abcam #ab16045), STAT3 (Cell Signaling Technology, #9139, #12640), pSTAT3 Tyr 705 (Cell Signaling Technology, #9145), SOCS3 (Cell Signaling Technology, #52113), β-actin (Sigma-Aldrich) and Histone H3 (Cell Signaling Technology, #4499). The bands were scanned and quantified as described [52].

### Constructs and lentiviral infection

Expression vectors encoding CypB and shRNA sequences of CypB and STAT3 were constructed and the lentivirus packaging was conducted as previously described [14]. The shRNA sequences were subcloned into the LV-12 (pGLVH6-CMV-LUC-2A-Puro-U6-shRNA) vector to generate a PVT1-shRNA lentivirus (shPVT1) (GenePharma) and the PVT1 cDNA was PCR amplified and subcloned into the LV-13 (pLenti-EF1a-LUC-F2A-Puro-CMV) vector (GenePharma) as described [53]. The infected cells were cultured in selection medium (culture medium with 1.5 μg/ml of puromycin) and collected for the downstream analysis at 2 weeks post-infection. All the sequences for targets are described in Supplementary Table S5.

### IHC and ISH

For IHC, the target molecules were performed on tissue microarray chips or mice tissue using CypB antibody (Abcam), phospho-STAT3antibody (Cell Signaling Technology) and Ki67 (Santa Cruz). For ISH, a 5′- and 3′-digoxigenin (DIG)-labeled locked nucleic acid-based probe specific for PVT1 (Exiqon) was incubated with the same tissue microarray chip. The results of IHC and ISH were independently scored by two independent observers. Expression levels were visualized and classified based on the percentage of positive cells and the intensity of staining as previously described [14, 54, 55]. The percentage of positive cells was divided into four grades (percentage cores): <1% (0), 1–25% (1), 26–50% (2), 51–75% (3) and >75% (4). The intensity of staining was divided into four grades (intensity scores): negative (0), weak (1), moderate (2), and strong (3). The histological score was determined by the following formula: overall scores = percentage score × intensity score. An overall score of 0–12 was calculated and graded as low (score: 0–4), high (score: 5–12).

### Cell proliferation assay

Target cells were seeded in 96-well plates (1 × 10^3^/well) at 48 h post-transfection. XTT assays were conducted to determine the cell growth according to the manufacturer’s instructions (Cell Proliferation Kit II (XTT), Roche) for 5 days. XTT labeling reagents were mixed and added to the wells following manufacture’s instruction. Varioskan Flash Multimode Reader (Thermo-Fisher) was used to read the absorbance at 466 nm with a reference wavelength at 650 nm.

### Flow cytometry analysis of cell cycle and apoptosis

Cells were seeded in 6-well plates at 2 × 10^5^ per well and harvested using trypsin. For cell cycle analysis, target cells were fixed in 75% ethanol and stained with propidium iodide (Sigma Aldrich) supplemented with RNase A (Roche) for 30 min at 22 °C 72 h post-transfection. The Annexin V-FITC Apoptosis Detection Kit (BD Biosciences) was used for apoptosis assays. Cells (1 × 10^4^) were starved in serum-free medium for 24 h, stained according to the manufacturer’s protocol and sorted using a fluorescence-activated cell sorting sorter (BD), and the data were analyzed using the Modfit software (BD).

### Transwell migration and invasion assays

For invasion assays, chamber inserts with an 8-μm pore size were first coated with 200 mg/mL Matrigel (Corning), and the uppermost chamber was plated with 1 × 10^5^ cells. For cell migration assays, the upper chamber with a noncoated membrane was plated with 5 × 10^4^ cells. Each assay was repeated three times, and three different inserts were used to obtain the mean number of cells in five fields per membrane as described [50].

### Mice, in vivo tumor growth and metastasis assays

For mice assays in Fig. 2A–D, 12 weeks-old female C57BL/6 mice were treated with normal diet or high fat diet (#D12108C, RDI, US). For the tumor xenograft mice model, 5 × 10^6^ cancer cells infected with lentiviral vectors or control were implanted into the flanks of 6-week-old female NOD/SCID (Server Combined Immune-deficiency) mice. The mice were sacrificed 20 days after injection, and tumors were collected and weighed. The tumor volume was calculated with the following formula: Tumor maximum diameter (L)×diameter along the perpendicular axis (W)2/2. For the in vivo metastasis assays, infected cells (5 × 10^6^ cells/100 μL of PBS) were injected into the tail vein or the spleen of NOD/SCID mice. The mice were sacrificed 4 weeks later, and the lungs and tumor tissues derived from various organs were dissected and examined as described [56]. All animals were housed and maintained in pathogen-free conditions. All animal studies complied with the Xi’an Jiaotong University animal use guidelines, and the protocol was approved by the University Animal Care Committee.

### Luciferase reporter assays

For luciferase assays, cells were transfected with appropriate plasmids in 24-well plates. Cells were harvested and lysed for luciferase assays 48 h after transfection. Luciferase assays were performed using a Dual-Luciferase Reporter Assay System (Promega, WI, USA) according to the manufacturer’s protocol. Firefly luciferase activity normalized to Renilla luciferase was used as an internal control. A site-directed mutagenesis kit (Agilent Technologies) was used to mutate the STAT3 binding sites of these vectors. The transfection experiments were performed in triplicate for each plasmid construct.

### Protein Immunofluorescence and fluorescence in situ hybridization (FISH)

Cells were plated onto glass coverslips and fixed with 4% paraformaldehyde for 20 min and permeabilized with 0.1% Triton X-100 in PBS for 15 min. Blocking solution was applied for 1 h at room temperature. Primary antibodies for CypB (Abcam #ab16045), STAT3 (Cell Signaling Technology, #12640) were applied at 4 °C overnight. FITC-conjugated and Cy3-conjugated secondary antibodies were loaded and incubated for 2 h at room temperature. Immunostaining signals and DAPI-stained nuclei were visualized and quantitation of CypB and STAT3 distribution was evaluated, as previously described [14]. The images were adjusted using the levels and brightness/contrast tools in Photoshop according to the guidelines for the presentation of digital data. FISH assays were performed with a FISH Kit (GenePharma, Shanghai, China) according to the manufacturer’s protocol. PVT1 probe labeled with CY3 was designed and synthesized by GenePharma Company. The cell nucleus was stained with DAPI. Signals were detected by confocal laser scanning microscopy.

### RNA isolation and RT-qPCR

Total RNA was extracted using a RNeasy Plus Mini Kit (Qiagen) according to the manufacturer’s instructions. cDNA was synthesized using a PrimeScript RT reagent kit (TaKaRa). SYBR Premix Ex Taq II (TaKaRa) was used to amplify the double-stranded cDNA of interest. RT-qPCR primers for the genes of interest were synthesized by TaKaRa. The levels of GAPDH were used as internal controls. A standard curve was established by amplifying diluted cDNA samples for calculation of relative target concentrations using Express SYBR GreenER qPCR SuperMix with Premixed ROX (Life Technologies). The 2–ΔΔCt method was used to determine the relative expression level of RNA between groups. The primer sequences used in this study are listed in Supplementary Table 5.

### RNA immunoprecipitation (RIP) and RIP-sequencing (RIPseq)

RIP was carried out with a Magna RIP™ RNA-Binding Protein Immunoprecipitation Kit (Millipore) with reference to the manufacturer’s instructions. Briefly, cells treated with IL-6 (50 ng/mL) or control for 24 h were harvested and then lysed in lysis buffer (50 mM Tris-HCl, pH = 7.4, 150 mM NaCl, 1% Triton-100, 0.1% SDS, 1.5 mM EDTA). Thereafter, cell lysates were incubated with RIP buffer containing magnetic beads. The beads were conjugated with the indicated antibody CypB (Abcam) or anti-IgG (Abcam) as a negative control. Then, the samples were digested by applying DNase I and proteinase K, and the immunoprecipitated RNA was isolated. Eventually, the enrichment of the purified RNAs was detected by RT-qPCR. The products were accurately quantified for sequencing applications using a quantitative real-time PCR (qRT-PCR)-based KAPA Biosystems Library Quantification Kit. Single-end sequencing (50 bp) was performed on a Angilent 4200 TapeStation.

### RNA pull-down

Full-length, serial truncations and antisense of PVT1 mRNA (#1, #2, #3, and #4 respectively) were transcribed with a HiScribe™ T7 Quick High Yield RNA Synthesis Kit (NEB) and purified with an RNeasy MinElute Cleanup Kit (QIAGEN), followed by labeling using an RNA 3′ End Desthiobiotinylation Kit (Thermo Fisher Scientific). Purified biotin-labeled RNA was heated and annealed to form a secondary structure, mixed with whole cell extract in RIP buffer for 1 h, and incubated with streptavidin agarose beads (Invitrogen) for 1 h. Finally, the RNA-binding proteins were analyzed by Western blot targeting CypB (Abcam).

### Ch-IP assay

ChIP assays were performed using the Magna ChIP G Assay kit (EMD Millipore). Cells were cross-linked with 1% formaldehyde for 10 min at room temperature and quenched in glycine. DNA was immunoprecipitated from the sonicated cell lysates using STAT3 antibody (Cell Signaling Technology) and subjected to PCR to amplify the STAT3 binding sites. The amplified fragments were then analyzed using agarose gel. A total of 10% of chromatin before immunoprecipitation was used as the input control, and a nonspecific antibody against IgG (BD) served as the negative control. PCR primers for Ch-IP are shown in Supplementary Table S5.

### Statistical analysis

SPSS software (version 19.0, SPSS Inc., Chicago, IL, USA) was used for statistical analyses. Continuous data were presented as the mean ± SD. and were compared between two groups by Student’s unpaired t-test. Frequencies of categorical variables were compared using the χ2 test. Spearman’s rank correlation coefficients were computed for assessing mutual association among clinical results. *P* < 0.05 was considered to be statistically significant (*P < 0.05, **P < 0.01).

## Supplementary information

Supplementary Figure S1-S8

Supplementary Figure S9

Supplementary Table S1-S5

Reproducibility checklist

## Data Availability

The datasets used and/or analyzed during the current study are available from the corresponding author upon reasonable request.
