# Supplementary material for: High-fat diet induced cyclophilin B enhances STAT3/lncRNA-PVT1 feedforward loop and promotes growth and metastasis in colorectal cancer
Source: Cell Death Dis. 2022 Oct 20;13(10):883. doi: 10.1038/s41419-022-05328-0 (PMC9584950; doi:10.1038/s41419-022-05328-0)
Supplement: Supplementary file 1 — Supplementary Figure S1-S8 [file 41419_2022_5328_MOESM1_ESM.pdf]

Supplementary Figure

Figure Legends.

Figure S1 Pan-Cancer analysis and GSEA analysis of CypB.

(A) CypB transcripts were increased by in most kinds of cancer using TCGA data. ACC, Adrenocortical carcinoma. BLCA, Bladder Urothelial Carcinoma. BRCA, Breast invasive carcinoma. CESC, Cervical squamous cell carcinoma and endocervical adenocarcinoma. CHOL, Cholangial carcinoma, COAD, Colon adenocarcinoma. DLBC, Lymphoid Neoplasm Diffuse Large B-cell Lymphoma. ESCA Esophageal carcinoma. GBM, Glioblastoma multiforme HNSC, Head and Neck squamous cell carcinoma. KICH, Kidney Chromophobe. KIRC, Kidney renal clear cell carcinoma. KIRP, Kidney renal papillary cell carcinoma. LAML, Acute Myeloid Leukemia. LGG, Brain Lower Grade Glioma. LIHC, Liver hepatocellular carcinoma. LUAD, Lung adenocarcinoma. LUSC, Lung squamous cell carcinoma, OV, Ovarian serous cystadenocarcinoma. PAAD, Pancreatic adenocarcinoma. PCPG, Pheochromocytoma and Paraganglioma. PRAD, Prostate adenocarcinoma. READ, Rectum adenocarcinoma. SARC Sarcoma. SKCM, Skin Cutaneous Melanoma. STAD, Stomach adenocarcinoma. TGCT, Testicular Germ Cell Tumors. THCA, Thyroid carcinoma. THYM, Thymoma. UCEC, Uterine Corpus Endometrial Carcinoma, UCS, Uterine Carcinosarcoma. UVM, Uveal Melanoma. Data were shown as transcripts per million (TPM). Green: Normal tissues. Red: Tumor tissues. (B-D) GSEA analysis of CypB in TCGA data of COAD and READ. Each Biological Process (B), Cellular Component (C) and Molecular Function (D) category is represented by a red, blue and green bar, respectively. The

height of the bar represents the number of IDs in the user list and also in the category.

Figure S2 GO analysis of CypB-binding lncRNAs. (A) Callpeaks of RIPseq results in HCT116 cells using CypB antibody. (B) GO analysis of CypB binding RNAs using RIPseq results targeting biological process (BP). (C) GO analysis of CypB binding RNAs using RIPseq results targeting molecular function (MF). (D) GO analysis of CypB binding RNAs using RIPseq results on cellular component (CC).

Figure S3 Screening of CypB interacting RNA. (A)Validation of 10 candidate lncRNAs binding to CypB protein by RIP-qPCR. (B) Overlap of predicted CypB-interacting lncRNAs by CatRapid database (http://s.tartaglialab.com, Z score ⩾0.2; Interaction propensity⩾20) and RNAseq results (foldchange ⩾1.5). (C) 3D-structure of CypB domain (PF000126) which were predicted to bind PVT1 (P1), generated on SWISS-MODEL (https://swissmodel.expasy.org/).

Figure S4 PVT1 promotes proliferation and decreases apoptosis. (A) Cell cycle analysis of Lenti-shPVT1-infected HCT116 and SW620 cells (left) and PVT1-vector infected Caco-2 and SW480 cells (right) using FACS. (B) Apoptosis analysis of Lenti-shPVT1-infected HCT116 and SW620 cells (left) and PVT1-vector infected Caco-2 and SW480 cells (right) using FACS. Apoptosis was induced by serum-free medium for 24h. Means ± SD of a representative experiment (n=3) performed in triplicates are shown. *, P<0.05; **, P<0.01; *N.S.*, not significant.

Figure S5 PVT1 promotes metastasis through CypB/STAT3 axis. (A) Immunoblots of CypB, pSTAT3 (Tyr705), and STAT3 in Caco-2 cells infected with PVT1 overexpressing vectors or control vectors, with or without co-infection of shCypB and

shSTAT3 lentiviral vectors. (B) Transwell assays showing migration and invasion of Caco-2 cells infected as in (A). Representative images of 3 independent biological replicates were shown. The data are presented as the means ± SDs. *, P<0.05; **, P<0.01; *N.S.*, not significant.

Figure S6 Knockdown of PVT1 decreases nuclear protein levels of CypB and STAT3.

(A) Nuclear protein were extracted and levels of CypB and STAT3 were detected by immunoblotting assays. Representative images of 3 independent biological replicates were shown. (B-C) Quantification of bands of CypB and STAT3 were conducted in QuantityOne software and Histone H3 were used as internal control. The data are presented as the means ± SDs. *, P<0.05; **, P<0.01; *N.S.*, not significant.

Figure S7 STAT3 promotes PVT1 transcription by binding to its promoter. (A) Schematic representation of mutation constructs spanning the − 2000 to + 500 region of the PVT1 promoter. Putative STAT3-binding sites in the PVT1 promoter are indicated in black, while mutated regions are indicated in red. (B) Luciferase activity of mutated PVT1 luciferase reporter constructs transfected into HCT116 cells with or without IL-6 treatment (followed by transfection at conc. 50ng/mL for 24h), Representative images of 3 independent biological replicates were shown. *, P<0.05; **, P<0.01; *N.S.*, not significant.

Figure S8 Positive correlation of CypB and PVT1 with STAT3-downstream interleukins in TCGA cohorts. (A-H) Correlation of PVT1 and CypB with STAT3-downstream targets using TCGA data of COAD and READ by GEPIA database

(http://gepia.cancer-pku.cn/). Y-axis, transcripts per million (TPM) of STAT3 downstream interleukins; X-axis, TPM of PVT1 and CypB. (A) Correlation of PVT1 with IL-1B (left, R=0.13, P=0.021) and correlation of CypB with IL-1B (right, R=0.22, P=3.8e-6); (B) Correlation of PVT1 with IL-6 (left, R=0.15, P=0.0028) and correlation of CypB with IL-6 (right, R=0.17, P=4e-4); (C) Correlation of PVT1 with IL-11 (left, R=0.27, P=3.5e-8) and correlation of CypB with IL-11 (right, R=0.3, P=3.3e-10); (D) Correlation of PVT1 with CCL3 (left, R=0.13, P=0.0083) and correlation of CypB with CCL3 (right, R=0.3, P=4.4e-10); (E) Correlation of PVT1 with CXCL8 (left, R=0.29, P=2.4e-9) and correlation of CypB with CXCL8 (right, R=0.38, P=6e-16); (F) Correlation of PVT1 with CXCL10 (left, R=0.3, P=3.3e-10) and correlation of CypB with CXCL10 (right, R=0.12, P=0.016); (G) Correlation of PVT1 with LIF (left, R=0.25, P=3.6e-7) and correlation of CypB with LIF (right, R=0.29, P=1.8e-9); (H) Correlation of PVT1 with OSM (left, R=0.26, P=4.4e-8) and correlation of CypB with OSM (right, R=0.3, P=2.3e-10).

Figure S9 Original images of immunoblots used in this manuscript.

Figure S1

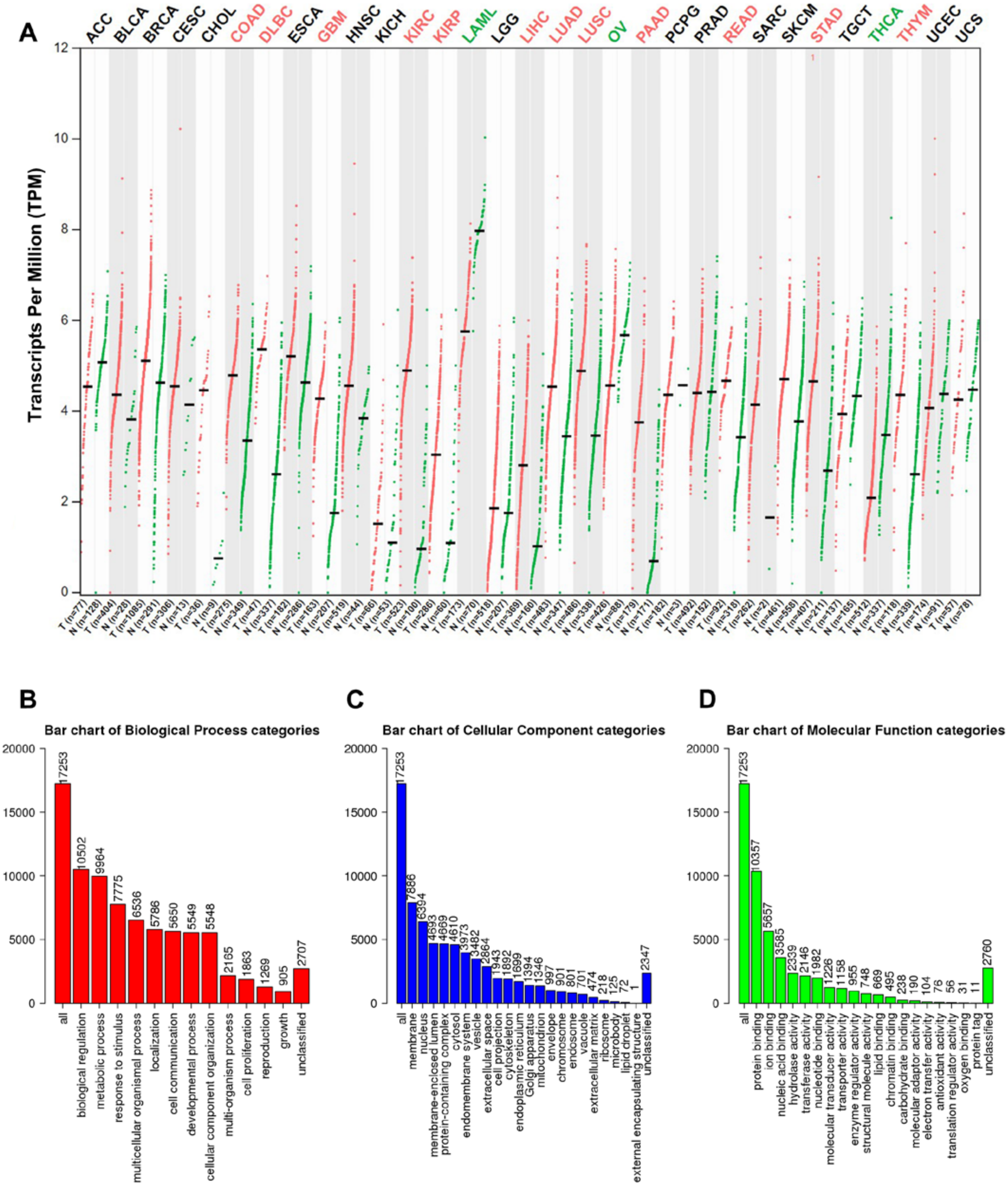
A
ACC
BLCA
BRCA
CESC
CHOL
COAD
DLBC
ESCA
GBM
HNSC
KICH
KIRC
KIRP
LAML
LGG
LIHC
LUAD
LUSC
OV
PAAD
PCPG
PRAD
READ
SARC
SKCM
STAD
TGCT
THCA
THYM
UCEC
UCS
Transcripts Per Million (TPM)
0
2
4
6
8
10
12
B
Bar chart of Biological Process categories
20000
15000
10000
5000
0
17253
10502
9964
7775
6536
5786
5650
5549
5548
2165
1863
1269
905
2707
all
biological regulation
metabolic process
response to stimulus
multicellular organismal process
localization
cell communication
developmental process
cellular component organization
multi-organism process
cell proliferation
reproduction
growth
unclassified
C
Bar chart of Cellular Component categories
20000
15000
10000
5000
0
17253
7886
6394
4693
4669
4610
3973
3482
2864
1943
1892
1699
1394
1346
997
901
801
701
474
218
125
72
1
2347
all
membrane
nucleus
membrane-enclosed lumen
protein-containing complex
cytosol
endomembrane system
vesicle
extracellular space
cell projection
cytoskeleton
endoplasmic reticulum
Golgi apparatus
mitochondrion
envelope
chromosome
endosome
vacuole
extracellular matrix
ribosome
microbody
lipid droplet
external encapsulating structure
unclassified
D
Bar chart of Molecular Function categories
20000
15000
10000
5000
0
17253
10357
5657
3585
2339
2146
1982
1226
1158
955
748
669
495
238
190
104
76
56
31
11
2760
all
protein binding
ion binding
nucleic acid binding
hydrolase activity
transferase activity
nucleotide binding
molecular transducer activity
transporter activity
enzyme regulator activity
structural molecule activity
lipid binding
chromatin binding
carbohydrate binding
molecular adaptor activity
electron transfer activity
antioxidant activity
translation regulator activity
oxygen binding
protein tag
unclassified

Figure S2

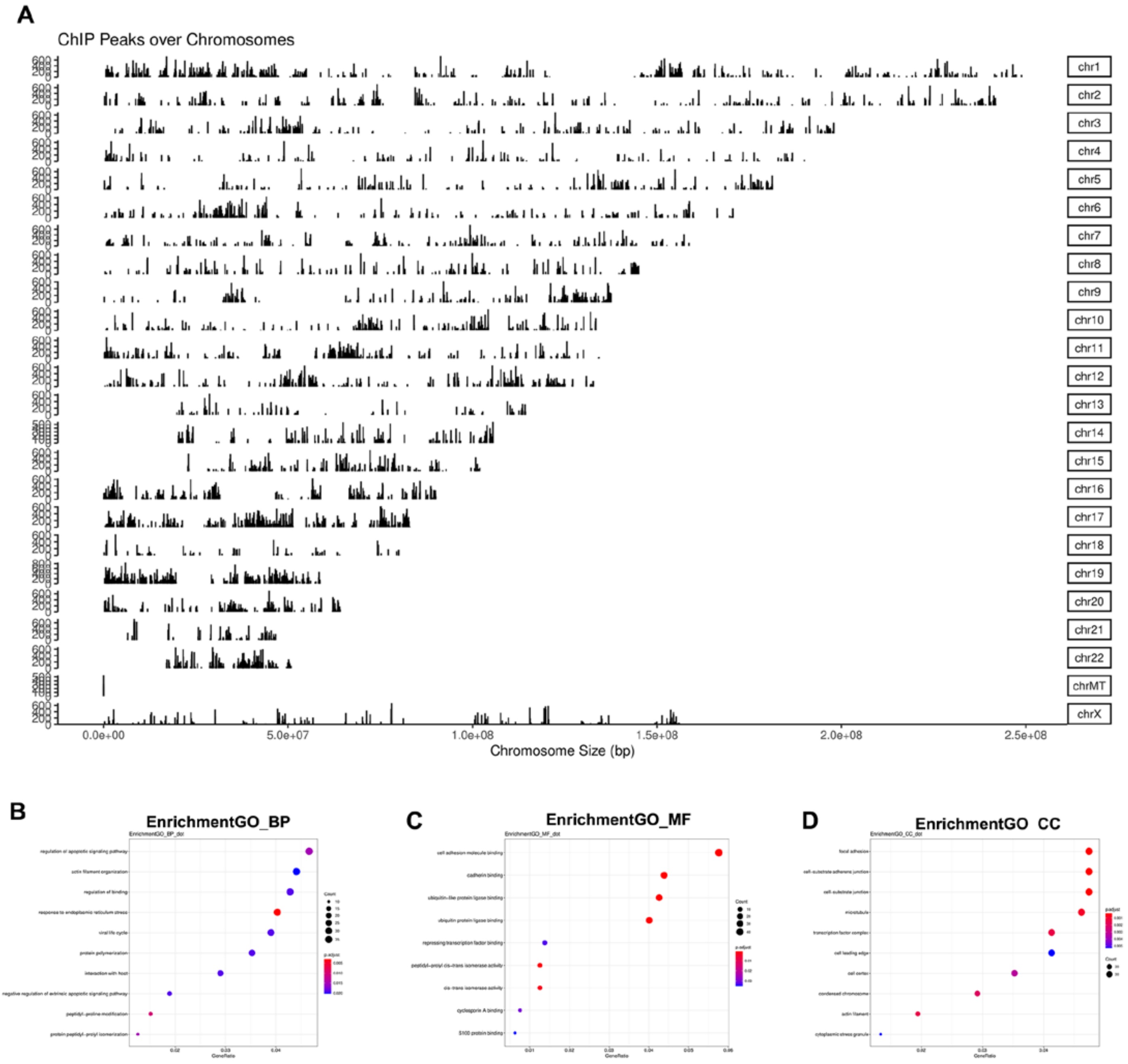
A
ChIP Peaks over Chromosomes
chr1
chr2
chr3
chr4
chr5
chr6
chr7
chr8
chr9
chr10
chr11
chr12
chr13
chr14
chr15
chr16
chr17
chr18
chr19
chr20
chr21
chr22
chrMT
chrX
0.0e+00
5.0e+07
1.0e+08
1.5e+08
2.0e+08
2.5e+08
Chromosome Size (bp)
B
EnrichmentGO_BP
regulation of apoptotic signaling pathway
actin filament organization
regulation of binding
response to endoplasmic reticulum stress
viral life cycle
protein polymerization
interaction with host
negative regulation of extrinsic apoptotic signaling pathway
peptidyl-proline modification
protein peptidyl-prolyl isomerization
GeneRatio
Count
p.adjust
C
EnrichmentGO_MF
cell adhesion molecule binding
cadherin binding
ubiquitin-like protein ligase binding
ubiquitin protein ligase binding
repressing transcription factor binding
peptidyl-prolyl cis-trans isomerase activity
cis-trans isomerase activity
cyclosporin A binding
S100 protein binding
GeneRatio
Count
p.adjust
D
EnrichmentGO CC
focal adhesion
cell-substrate adherens junction
cell-substrate junction
microtubule
transcription factor complex
cell leading edge
cell cortex
condensed chromosome
actin filament
cytoplasmic stress granule
GeneRatio
p.adjust
Count

Figure S3

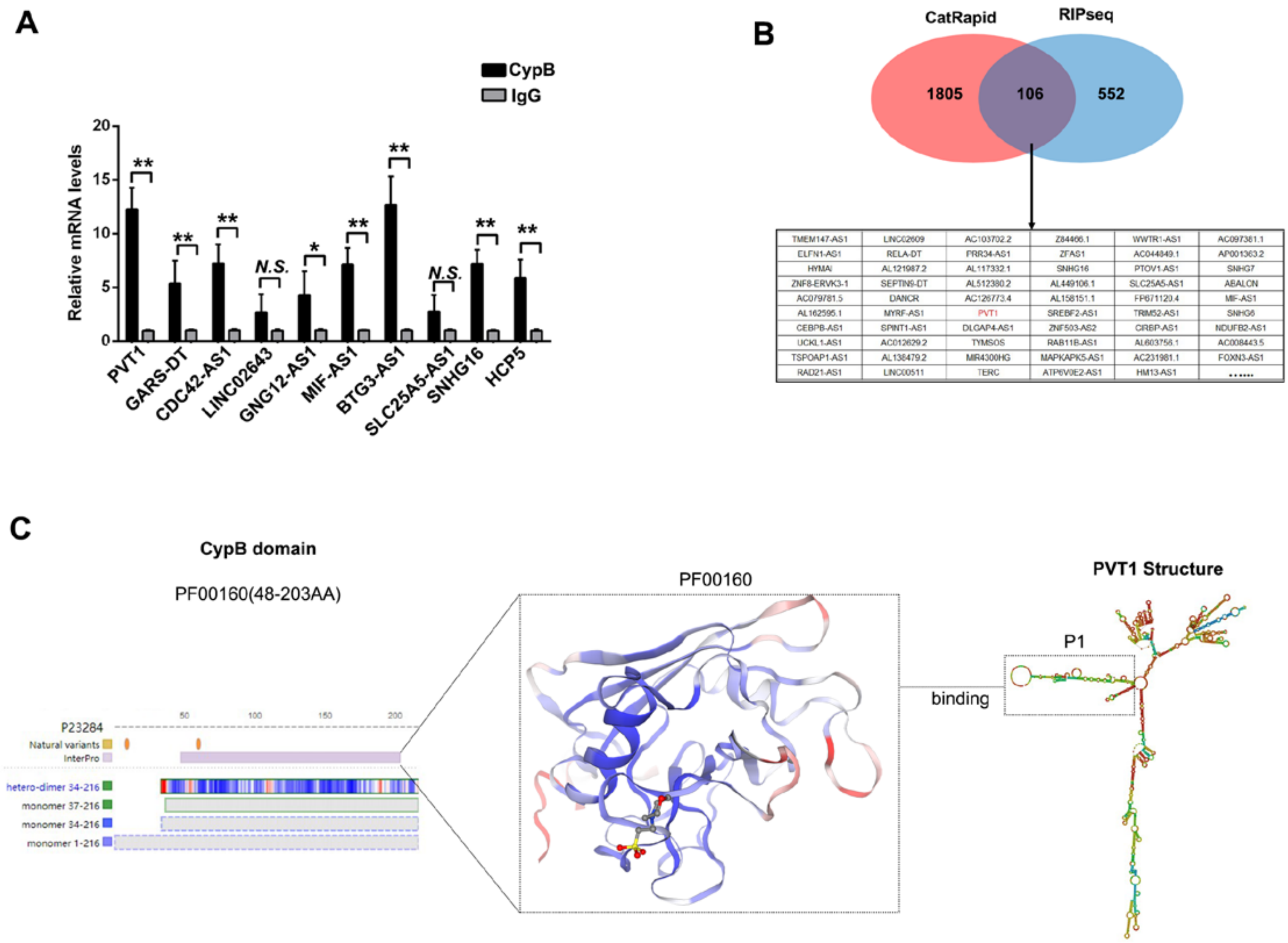

TMEM147-AS1	LINC02609	AC103702.2	Z84466.1	WWTR1-AS1	AC097381.1
ELFN1-AS1	RELA-DT	PRR34-AS1	ZFAS1	AC044849.1	AP001363.2
HYMAI	AL121987.2	AL117332.1	SNHG16	PTOV1-AS1	SNHG7
ZNF8-ERVK3-1	SEPTIN9-DT	AL512380.2	AL449106.1	SLC25A5-AS1	ABALON
AC079781.5	DANCR	AC126773.4	AL158151.1	FP671120.4	MIF-AS1
AL162595.1	MYRF-AS1	PVT1	SREBF2-AS1	TRIM52-AS1	SNHG6
CEBPB-AS1	SPINT1-AS1	DLGAP4-AS1	ZNF503-AS2	CIRBP-AS1	NDUFB2-AS1
UCKL1-AS1	AC012629.2	TYMSOS	RAB11B-AS1	AL603756.1	AC008443.5
TSPOAP1-AS1	AL138479.2	MIR4300HG	MAPKAPK5-AS1	AC231981.1	FOXN3-AS1
RAD21-AS1	LINC00511	TERC	ATP6V0E2-AS1	HM13-AS1	......

Figure S4

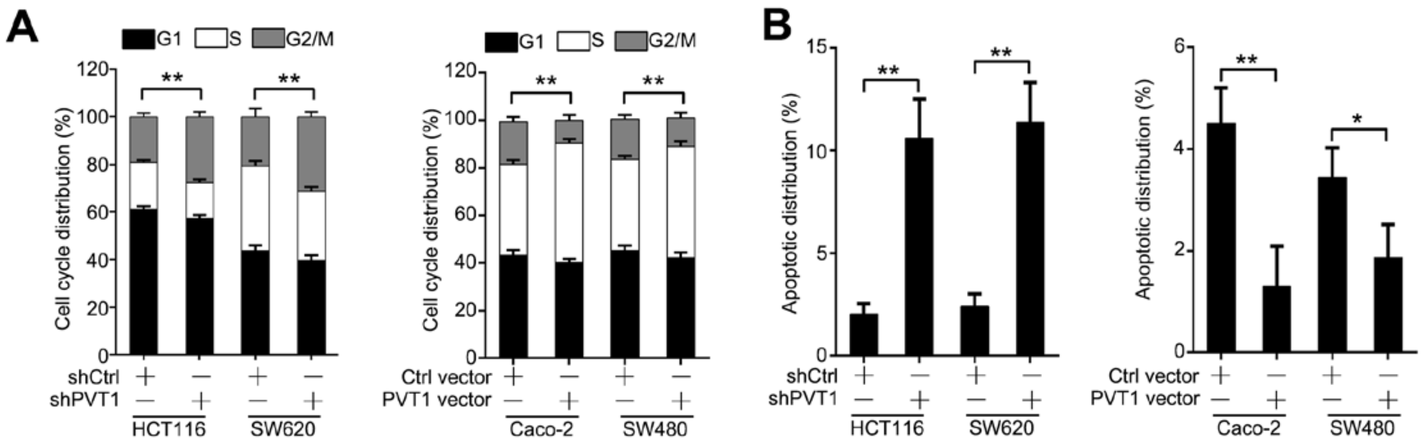
A
G1
S
G2/M
Cell cycle distribution (%)
**
shCtrl
shPVT1
HCT116
SW620
Ctrl vector
PVT1 vector
Caco-2
SW480
B
Apoptotic distribution (%)
*

Figure S5

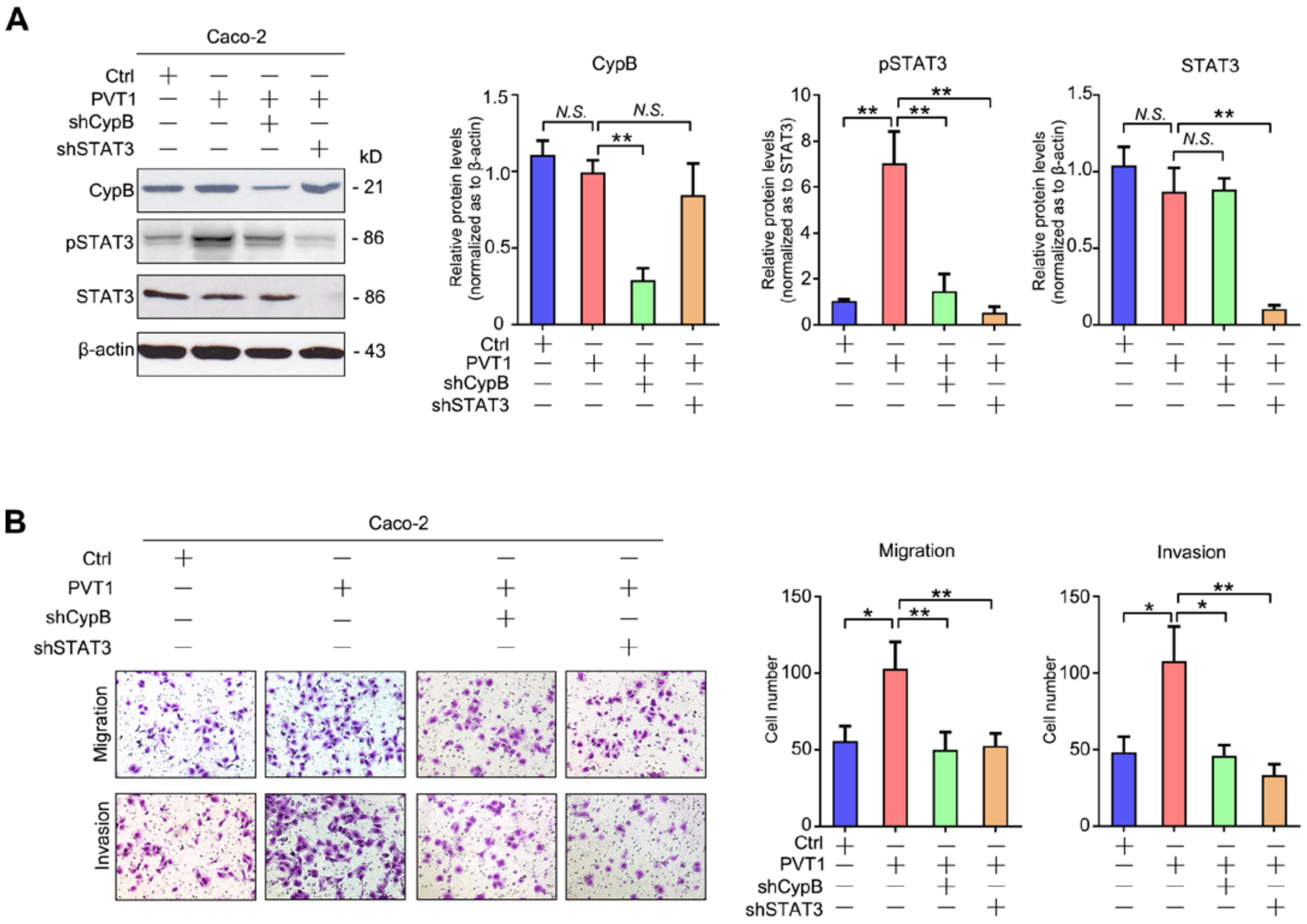
A
Caco-2
Ctrl
PVT1
shCypB
shSTAT3
kD
CypB
pSTAT3
STAT3
β-actin
- 21
- 86
- 86
- 43
CypB
pSTAT3
STAT3
Relative protein levels (normalized as to β-actin)
Relative protein levels (normalized as to STAT3)
N.S.
**
B
Caco-2
Migration
Invasion
Cell number
*
**

Figure S6

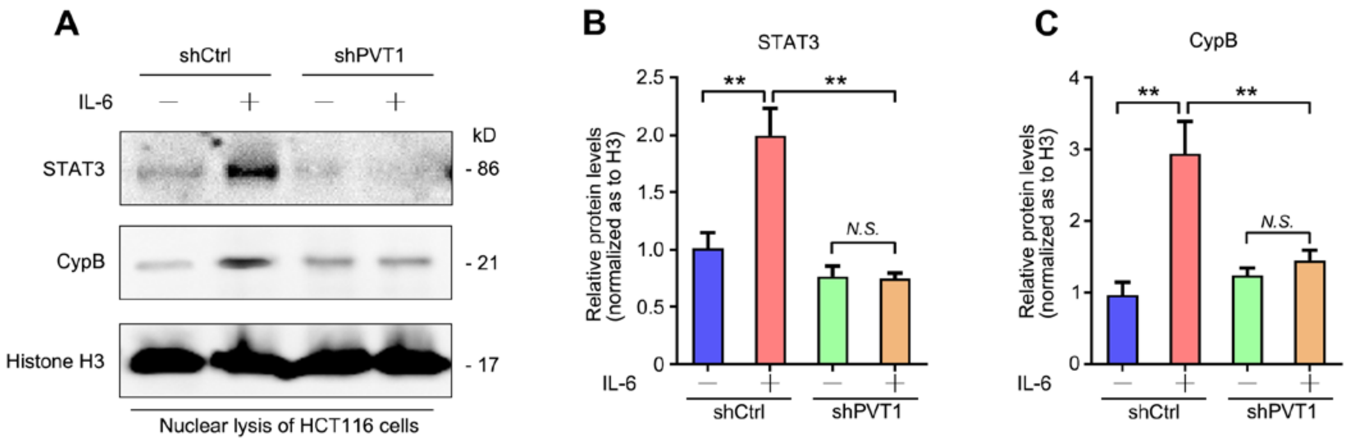
A
shCtrl
shPVT1
IL-6
kD
STAT3
- 86
CypB
- 21
Histone H3
- 17
Nuclear lysis of HCT116 cells
B
STAT3
Relative protein levels (normalized as to H3)
**
N.S.
IL-6
shCtrl
shPVT1
C
CypB
Relative protein levels (normalized as to H3)
**
N.S.
IL-6
shCtrl
shPVT1

Figure S7

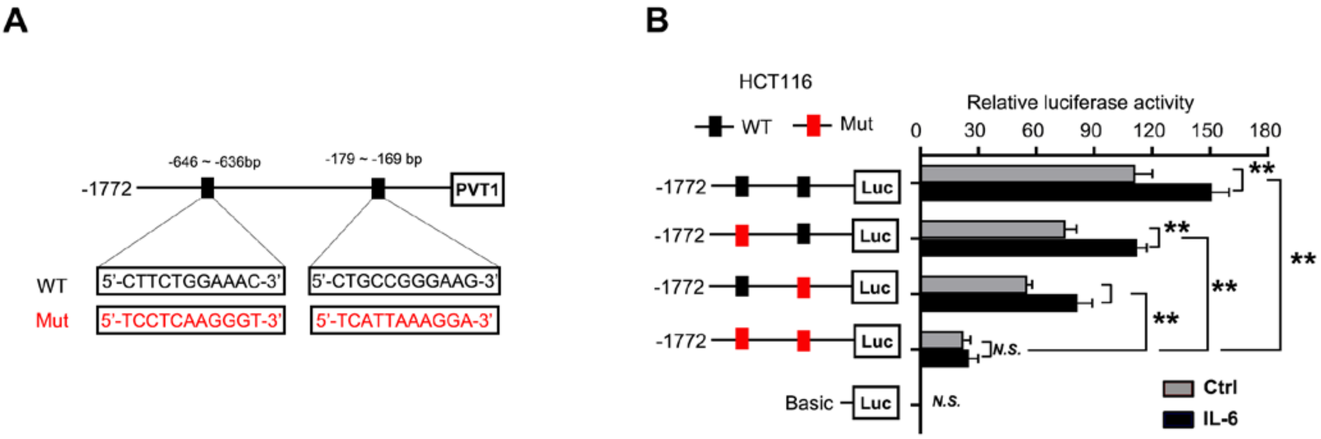

A
-646 ~ -636bp
-179 ~ -169 bp
-1772
PVT1
WT
Mut
5'-CTTCTGGAAAC-3'
5'-CTGCCGGGAAG-3'
5'-TCCTCAAGGGT-3'
5'-TCATTAAAGGA-3'
B
HCT116
WT
Mut
Relative luciferase activity
0 30 60 90 120 150 180
-1772
Luc
Basic
**
N.S.
Ctrl
IL-6

Figure S8

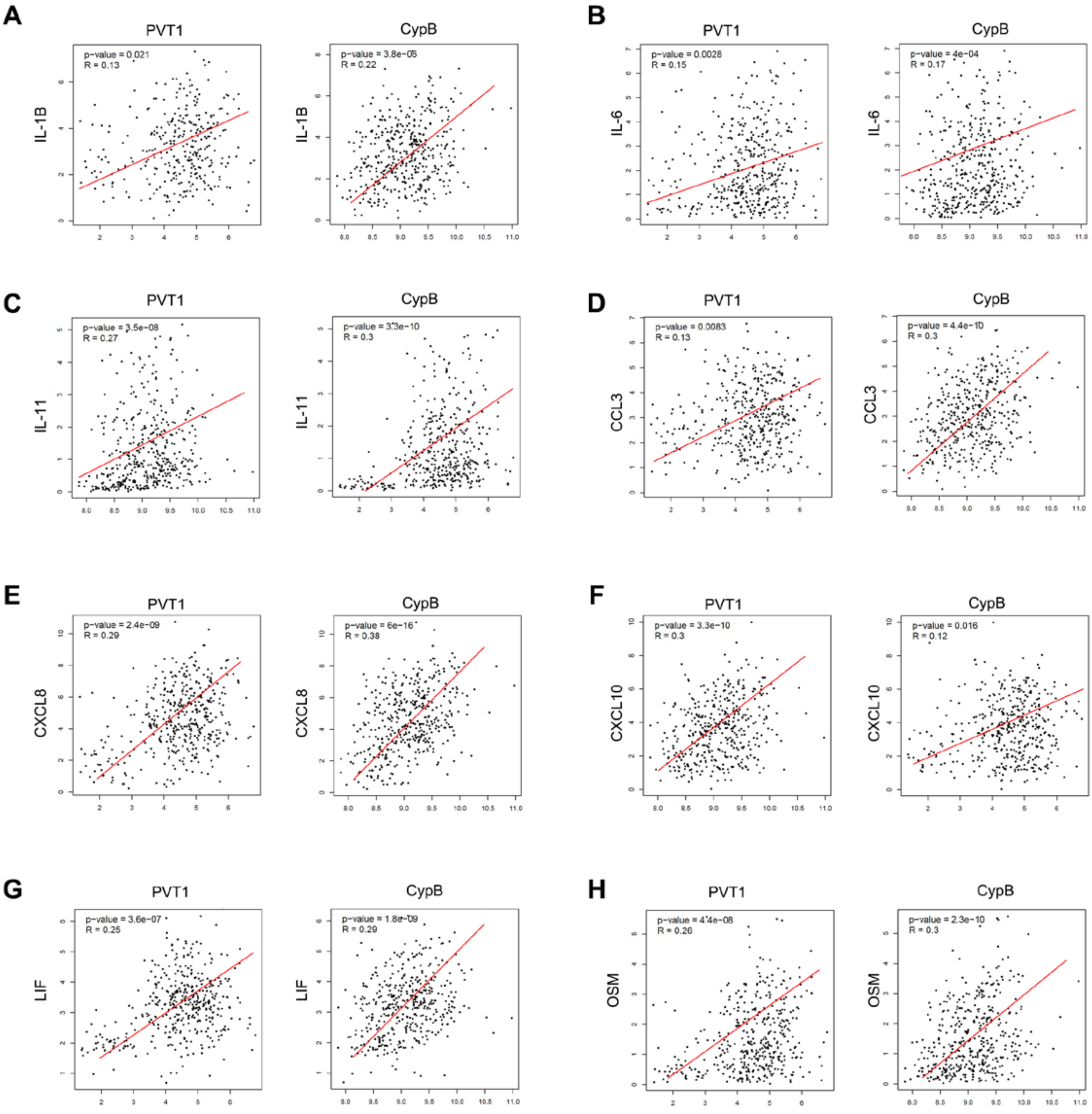
A
PVT1
p-value = 0.021
R = 0.13
IL-1B
CypB
p-value = 3.8e-06
R = 0.22
IL-1B
B
PVT1
p-value = 0.0028
R = 0.15
IL-6
CypB
p-value = 4e-04
R = 0.17
IL-6
C
PVT1
p-value = 3.5e-08
R = 0.27
IL-11
CypB
p-value = 3.3e-10
R = 0.3
IL-11
D
PVT1
p-value = 0.0083
R = 0.13
CCL3
CypB
p-value = 4.4e-10
R = 0.3
CCL3
E
PVT1
p-value = 2.4e-09
R = 0.29
CXCL8
CypB
p-value = 6e-16
R = 0.38
CXCL8
F
PVT1
p-value = 3.3e-10
R = 0.3
CXCL10
CypB
p-value = 0.016
R = 0.12
CXCL10
G
PVT1
p-value = 3.6e-07
R = 0.25
LIF
CypB
p-value = 1.8e-09
R = 0.29
LIF
H
PVT1
p-value = 4.4e-08
R = 0.26
OSM
CypB
p-value = 2.3e-10
R = 0.3
OSM
