# Supplementary material for: High-fat diet induced cyclophilin B enhances STAT3/lncRNA-PVT1 feedforward loop and promotes growth and metastasis in colorectal cancer
Source: Cell Death Dis. 2022 Oct 20;13(10):883. doi: 10.1038/s41419-022-05328-0 (PMC9584950; doi:10.1038/s41419-022-05328-0)

Figure S9

Figure 1H

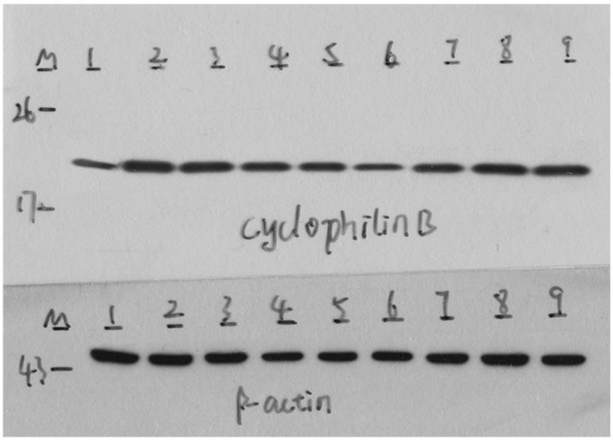

Figure 2A left

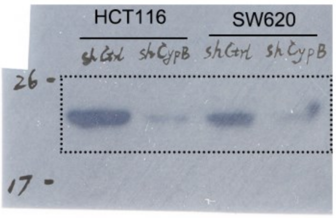

CypB

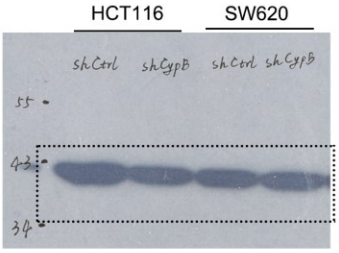

β-actin

Figure 2A right

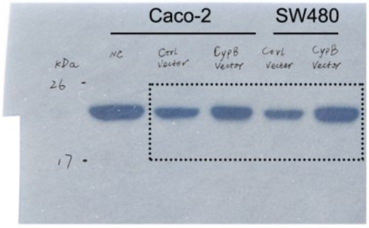

CypB

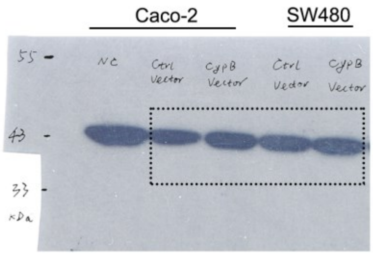

β-actin

Figure 4F

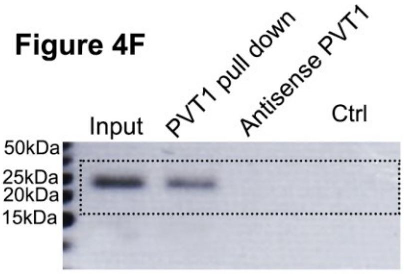

CypB

Figure 4I

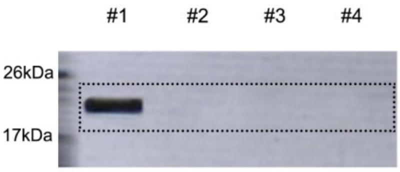

CypB

Figure 6D

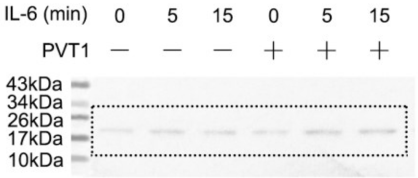

CypB

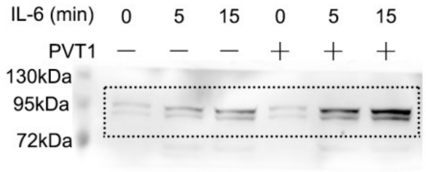

pSTAT3

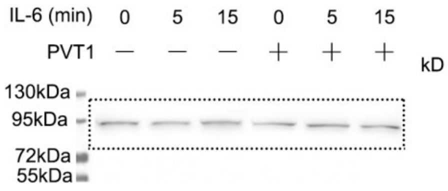

STAT3

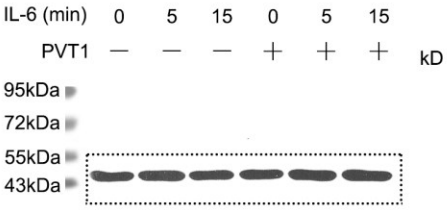

β-actin

Figure 6G

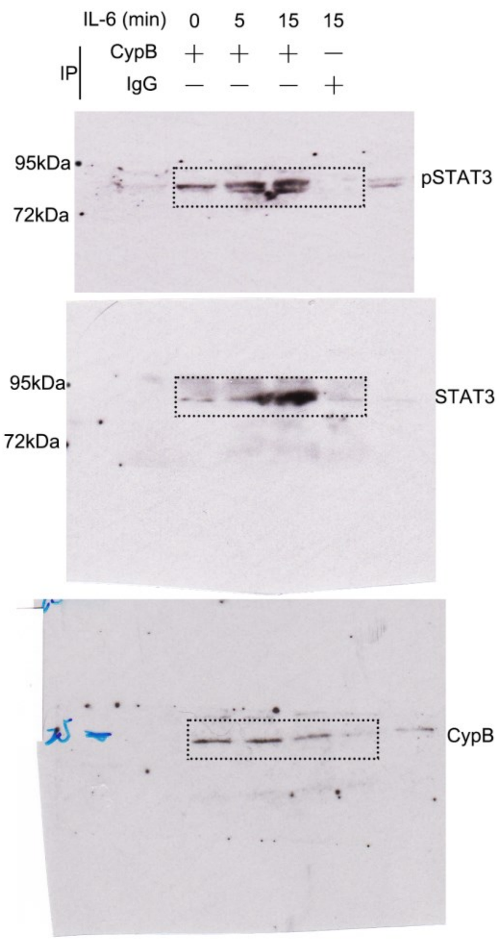

Figure 6H

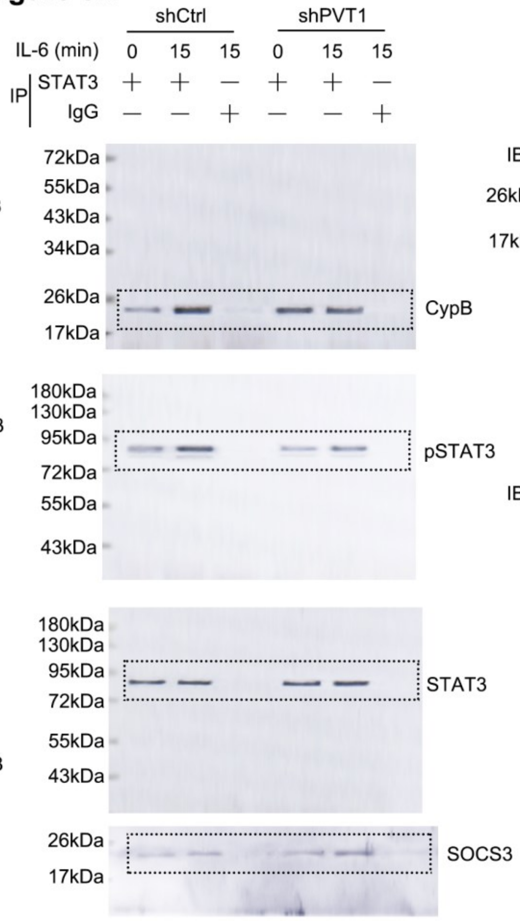

Figure 6I

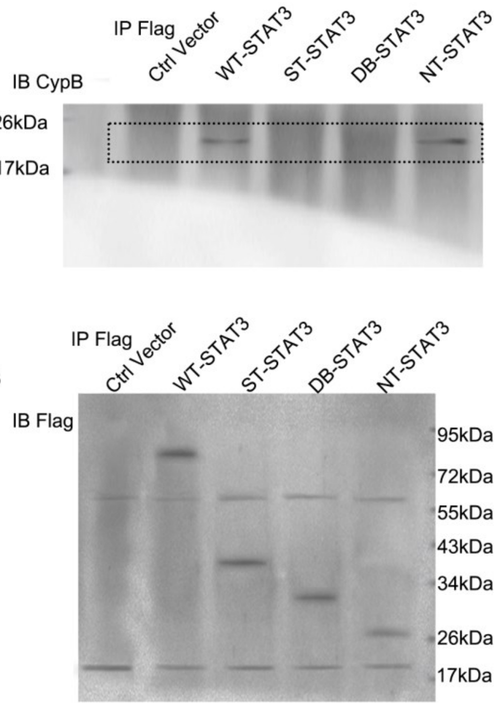

Figure S5A

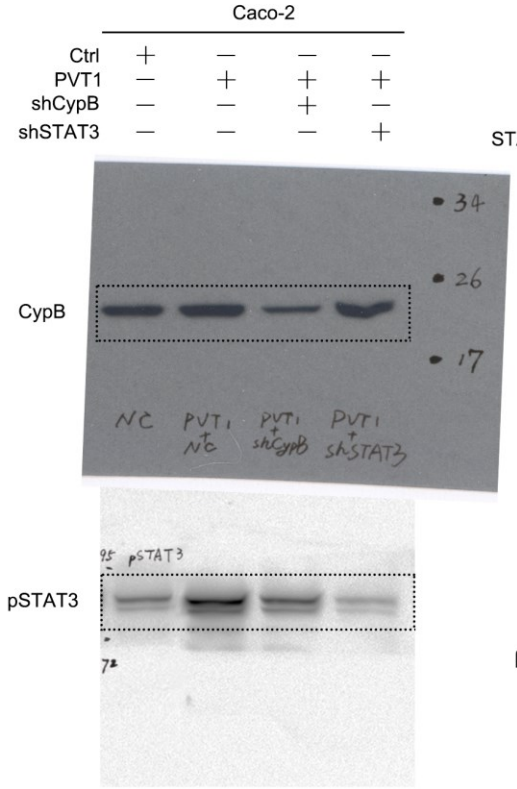

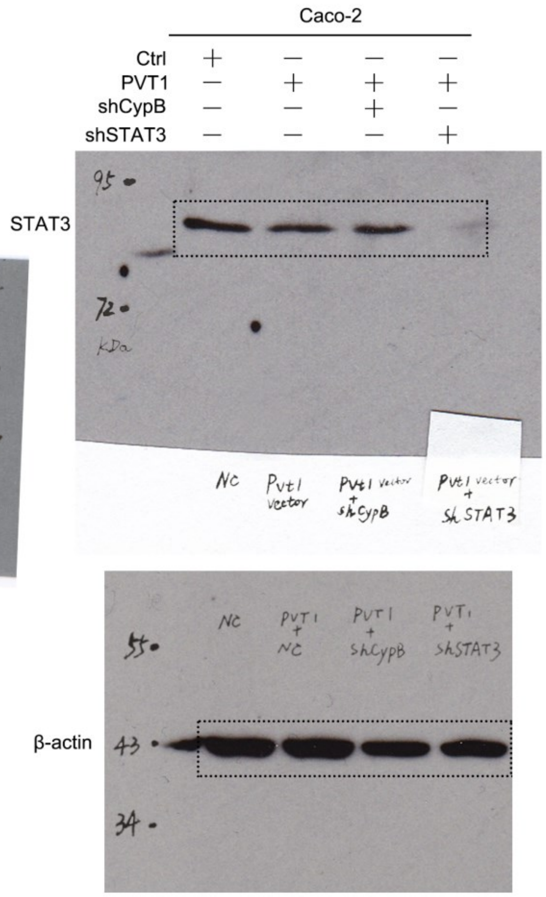

Figure S6A

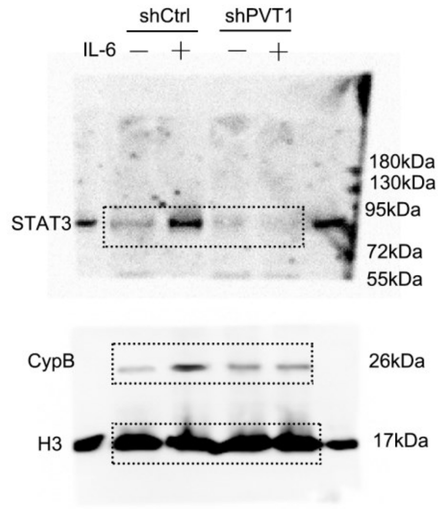

Supplement: Supplementary file 2 — Supplementary Figure S9 [file 41419_2022_5328_MOESM2_ESM.pdf]
