# Supplementary material for: High-fat diet induced cyclophilin B enhances STAT3/lncRNA-PVT1 feedforward loop and promotes growth and metastasis in colorectal cancer
Source: Cell Death Dis. 2022 Oct 20;13(10):883. doi: 10.1038/s41419-022-05328-0 (PMC9584950; doi:10.1038/s41419-022-05328-0)
Supplement: Supplementary file 3 — Supplementary Table S1-S5 [file 41419_2022_5328_MOESM3_ESM.pdf]

Table S1. CypB expression in CRC tissue microarray.

	cases	CypB				P value
		-	+	++	+++	
Adjacent	80	21	52	7	0	
Cancer	80	16	38	19	7	0.0015#
Metastatic lymph nodes	80	13	16	27	24	＜0.001*

Frequencies of categorical variables were compared using the χ2 test.
Cancer versus Adjacent tissues; * Metastatic lymph nodes versus cancer

Table S2 Correlations of Clinico-pathological variables with CypB expression of TMA cohort.

Clinicopathological Feature	No. of Patients	Percent	CypB levels Low (n=37)	CypB levels High (n=43)	P value
Gender					
Male	56	70.00	24	32	0.4638
Female	24	30.00	13	11	
Age					
<60	32	40.00	14	18	0.3662
>60	48	60.00	23	25	
Tumor Size					
≤5 cm	36	45.00	22	14	0.1596
>5 cm	44	55.00	15	29	
Depth of Invasion					
T1	5	6.25	4	1	0.0138
T2	15	18.75	11	4	
T3	46	57.50	19	27	
T4	14	17.50	3	11	
Tumor stage					
I - II	33	41.25	23	10	0.0004
III - IV	47	58.75	14	33	
Tumor differentiation					
Well or moderate	45	56.25	24	21	0.1496
Poor	35	43.75	13	22	
Organ metastasis					
Negative	49	61.25	29	20	0.0035
Positive	31	38.75	8	23	

Frequencies of categorical variables were compared using the $\chi 2$ test.

Table S3 Univariate and multivariate analysis for disease free-overall survival.

Factors	Univariate analysis			Multivariate analysis[2]		
	HR	95%CI	P	HR	95%CI	P
Gender (male/female)	1.170	0.604-2.267	0.642	-	-	-
Age (>60/<60)	1.035	0.569-1.881	0.561	-	-	-
Tumor Size (≤5 cm/>5 cm)	0.504	0.272-0.934	0.030	0.923	0.757-1.098	0.183
Depth of Tumor (T1-T2/T3-T4)	0.106	0.033-0.345	<0.0001	0.226	0.065-0.789	0.020
Tumor Stage (I-II/III-IV)	0.265	0.131-0.538	<0.0001	0.469	0.226-0.973	0.042
Differentiation (Well/Moderate - Poor)	0.423	0.233-766	0.005	0.480	0.211-1.093	0.080
Organ metastasis (Negative/Positive)	0.127	0.064-0.255	<0.0001	0.211	0.102-0.436	<0.0001
CypB expression (low/high)	0.305	0.161-0.578	<0.0001	0.374	0.194-0.719	0.003

[1] Analysis was conducted on 80 cases shown in Table S2. Hazard ratios (95% confidence interval [CI]) and p-values were calculated using univariate or multivariate Cox proportional hazard regression.

[2] Multivariate analysis performed only for variables significant in the univariate analysis.

Table S4 Potential STAT3 binding site on PVT1 promoter (-2000bp-500bp)[#].

Score	Relative score	Start	End	Strand	Predicted site sequence
10.627	0.926296098096034	9	19	-1	CTGCATGGAAA
9.785	0.9160956434948	141	151	1	GTTTCTGGAAA
5.342	0.862270679369763	218	228	-1	ATGATGAGAAA
4.951	0.857533888693893	832	842	-1	TTGCTGGGATT
10.619	0.926199181662768	1355	1365	1	CTTCTGGAAAC
4.648	0.853863178783947	1576	1586	1	CTTCCAGGACG
14.105	0.968430517458375	1822	1832	1	CTGCCGGGAAG
7.119	0.883798242108946	2147	2157	1	CTTCTGTGAAG
5.460	0.863700196760434	2209	2219	1	TGGCTGGGAAG
4.334	0.850059208778262	2302	2312	-1	GGTCCTGGAAG

[#]Predicted on JASPAR (https://jaspar.genereg.net/). 3 additional sites on the same region were not shown in this table (2 at 141-151 and 1 at 1576-1586).

Table S5 Sequences for shRNAs of targets and PCR primers used in this study.

shRNAs or PCR targets	Sequence
**shRNAs**	
shCypB	5’-GGTGGAGAGCACCAAGACA-3’
shSTAT3	5’-CCGUGGAACCAUACACAAA dTdT-3’
shPVT1	5'-CAGCUGGGCUUGAGAUUCCUGGGAA-3'
**Primers for PVT1 promoter construct**	
-2000~+500bp	5'cgacgcgtGGGGAGATTTCCATGCAGAAGGGACTGGG 3'
-1772~+500bp	5'cgacgcgtCTTCTTTTATATTTAAAAGATGGATGA 3'
-1158~+500bp	5'cgacgcgtTTTGGGAGGCCGAAGCGAGTGGATCATCT 3'
-635~+500bp	5'cgacgcgtAGCGGGAGACGTCGAAGGAGGCGAGAAG 3'
-414~+500bp	5'cgacgcgtGTGTGGGGAAGGGTAAGAGGGCTCAGGGA 3'
-168~+500bp	5'cgacgcgtCAGGCTGAGGGGCGCACCGGGCGGCG 3'
+313~+500bp	5'cgacgcgtCTGAGGATTTCAGCTCTACCCATGGGC 3'
Basic	5'ccgctcgagACTGGGGAGAATCGCTTGACCCCGGGAGGC 3'
**Ch-IP primers**	
Ch-IP NC	Forward：5'-TTGGCTTCCTGTCACCTCAG- 3'<br>Reverse：5'-TTATAATTCTCACTTGTCAAG- 3'
Ch-IP 1	Forward：5'-CCAGCATCCCCCAGGAGGACAC-3'<br>Reverse：5'-CAGGTGCCTGAGCTGCAGTTG-3'
Ch-IP 2	Forward：5'-GCATTTAAGGGACTCGTTGAAC-3'<br>Reverse：5'-TGGTGAGAAGGGCGCGCGCAC-3'
Ch-IP 3	Forward：5'-CCGGCCCGGTGGGACTCCGG-3'<br>Reverse：5'-TCCCCAGGTCCCCGCCCGCCG-3'
**PCR primers**	
CypB	Forward: 5’-TGTGGCCTTAGCTACAGGAG -3’<br>Reverse : 5’-CCAGGCCCGTAGTGCTTCAG-3’
il-6	Forward: 5'-GCAAGAGACTTCCATCCAGT-3'<br>Reverse： 5'-CATGTACTCCAGGTAGCTAT-3'
tnf-α	Forward: 5’-GACCCTCACACTCAGATCATCT -3’<br>Reverse：5’-CCTCCACTTGGTGGTTTGCT-3’
PVT1	Forward: 5’-CAACAGGAGGACAGCTTCAA-3’<br>Reverse: 5′-GGCGATGAAGTTCGTACTCA-3′
GARS-DT	Forward: 5'-AGCAGCAAAGCCTGTCATCT-3’<br>Reverse: 5'-CCCCTGAAAGCCAAATCGGA-3’

CDC42-AS1	Forward: 5'-TCTCGTGGAATCTGGACCTG-3’<br>REVERSE: 5'-GCACGATTCTCTAGCAAGGC-3’
LINC02643	Forward: 5'-CGCGATCTGGACCAATCAGA-3’<br>Reverse: 5'-GCCTAGACCACTGGAATGAGC-3’
GNG12-AS1	Forward: 5'-CGGAACCTGCGGATACAGAG-3’<br>Reverse: GACAAACAGGGACCACACGA-3’
MIF-AS1	Forward: 5’-ACATCGGCATGATGGCAGAA-3’<br>Reverse: 5′-TCACAAAAGGCGGGACCAC-3’
BTG3-AS1	Forward: 5'-TTCCCTCCCCGGCAAAAAG-3’<br>Reverse: 5'- GAGACAGCCGAAAGGACCAA-3’
SLC25A5-AS1	Forward: 5′-ACGGAAC TTGTGATTACACTAT-3′,<br>Reverse: 5′-CCTTCACTGGGTAAGCATT-3′;
SNHG16	Forward: 5′-CAGAATGCCATGGTTTCCCC-3′<br>Reverse: 5′-TGGCAAGAGACTTCCTGAGG-3′
HCP5	Forward: 5′-TATCCCTGTGAAGATGAACC-3′<br>Reverse: 5′-TGCCACCTCTAAATGTCCTA-3′
GAPDH	Forward: 5’-GGGAAACTGTGGCGTGAT-3’<br>Reverse: 5′-GAGTGGGTGTCGCTGTTGA-3′
c-Myc	Forward: 5’-GGACTTGTTGCGGAAACGAC-3’<br>Forward: 5’-CTCAGCCAAGGTTGTGAGGT-3’
CCND1	Forward: 5’: CGATGCCAACCTCCTCAACG-3’<br>Reverse: 5′-CCAGGTAGTTCA TGGCCAGC-3’
Bcl2	Forward:5’-GATGTGATGCCTCTGCGAAG-3’<br>Reverse: 5’-CATGCTGATGTCTCTGGAATCT-3’
BCL-xl	Forward: 5′-CTG AAT CGG AGA TGG AGA CC-3′<br>Reverse: 5′-TGG GAT GTC AGG TCA CTG AA-3′
Survivin	Forward: 5’- AGCCCTTTCTCAAGGACCAC-3’<br>Reverse: 5’- CAGCTCCTTGAAGCAGAAGAA-3
Twist1	Forward: 5'-GCCAGGTACATCGACTTCCTCT-3'<br>Reverse: 5'-TCCATCCTCCAGACCGAGAAGG-3'
